# Genomic variation across distribution of Micro-Tom, a model cultivar of tomato (*Solanum lycopersicum*)

**DOI:** 10.1093/dnares/dsae016

**Published:** 2024-06-07

**Authors:** Hideki Nagasaki, Kenta Shirasawa, Ken Hoshikawa, Sachiko Isobe, Hiroshi Ezura, Koh Aoki, Hideki Hirakawa

**Affiliations:** Department of Applied Genomics, Kazusa DNA Research Institute, 2-6-7 Kazusa-kamatari, Kisarazu, Chiba 292-0818, Japan; Department of Frontier Research and Development, Kazusa DNA Research Institute, 2-6-7 Kazusa-kamatari, Kisarazu, Chiba 292-0818, Japan; Tsukuba Plant Innovation Research Center, Institute of Life and Environmental Sciences, University of Tsukuba, Tsukuba 305-8572, Japan; Biological Resources and Post-harvest Division, Japan International Research Center for Agricultural Sciences, 1-1 Ohwashi, Tsukuba, Ibaraki 305-8686, Japan; Department of Applied Genomics, Kazusa DNA Research Institute, 2-6-7 Kazusa-kamatari, Kisarazu, Chiba 292-0818, Japan; Tsukuba Plant Innovation Research Center, Institute of Life and Environmental Sciences, University of Tsukuba, Tsukuba 305-8572, Japan; Graduate School of Life and Environmental Sciences, Osaka Metropolitan University, 1-1 Gakuen-cho, Naka-ku, Sakai, Osaka 599-8531, Japan; Department of Applied Genomics, Kazusa DNA Research Institute, 2-6-7 Kazusa-kamatari, Kisarazu, Chiba 292-0818, Japan

**Keywords:** *Solanum lycopersicum*, Micro-Tom, polymorphism, genome diversity, next-generation sequencing

## Abstract

Micro-Tom is a cultivar of tomato (*Solanum lycopersicum*), which is known as a major crop and model plant in Solanaceae. Micro-Tom has phenotypic traits such as dwarfism, and substantial EMS-mutagenized lines have been reported. After Micro-Tom was generated in Florida, USA, it was distributed to research institutes worldwide and used as a genetic resource. In Japan, the Micro-Tom lines have been genetically fixed; currently, three lines have been re-distributed from three institutes, but many phenotypes among the lines have been observed. We have determined the genome sequence *de novo* of the Micro-Tom KDRI line, one of the Micro-Tom lines distributed from Kazusa DNA Research Institute (KDRI) in Japan, and have built chromosome-scale pseudomolecules. Genotypes among six Micro-Tom lines, including three in Japan, one in the United States, one in France, and one in Brazil showed phenotypic alternation. Here, we unveiled the swift emergence of genetic diversity in both phenotypes and genotypes within the Micro-Tom genome sequence during its propagation. These findings offer valuable insights crucial for the management of bioresources.

## 1. Introduction

Tomato (*Solanum lycopersicum*) is a major crop cultivated around the world. Its fruits have excellent properties as a low-calorie food containing various nutrients such as antioxidant-rich soluble phenolic compounds and hydrophilic vitamin C, along with lipophilic compounds such as vitamin E, lutein, and carotenoids like lycopene.^1^ Currently, there are thought to be thousands of varieties worldwide,^2^ with differences in fruit size, shape, colour intensity, firmness, and so on among them.^3^ Micro-Tom is a tomato cultivar that inherits many biological characteristics from tomato families and has some different phenotypic advantages such as small plant size (20–30 cm in height) and a short life cycle (3–4 months).^4^ It is, therefore, used as model tomato plant.

**Figure 1.**
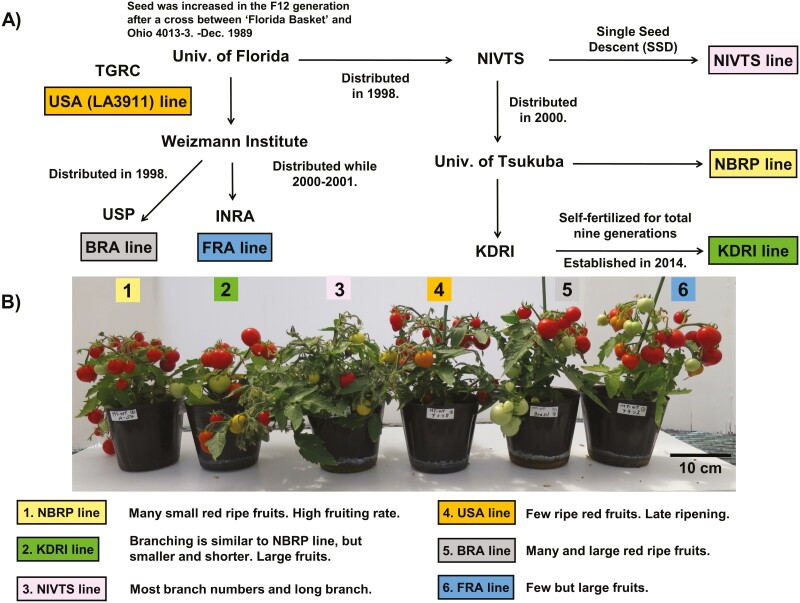
Worldwide propagation path and comparison of phenotypes among Micro-Tom lines. (A) Worldwide propagation of the Micro-Tom lines. The original Micro-Tom line, LA3911 (USA line), was developed by John W. Scott at the University of Florida. LA3911 was distributed from the Tomato Genetics Resource Center (TGRC) at UC Davis (USA line) to INRA in France (FRA line) and Lázaro Peres of Universidade de São Paulo (USP) in Brazil through Weizmann Institute in Israel. The right side indicates the development and distribution in Japan (NIVTS line at the NARO Institute of Vegetable and Tea Science, the NBRP line at the University of Tsukuba, and the KDRI line at the Kazusa DNA Research Institute). (B) Comparison of phenotypes among six Micro-Tom lines. Micro-Tom lines cultivated 104 days after sowing (except the NBRP line, which was cultivated 90 days after sowing) in a green house and examples of phenotypes are shown. The details on the phenotypes among the six Micro-Tom lines are described in Supplementary Data 1 Table 2.

Micro-Tom was originally generated by a crossing ‘Florida Basket’ and ‘Ohio 4013-3’ and bred at the University of Florida.^5^ F_12_ generation was propagated by and distributed from the Tomato Genetics Resource Center (TGRC) at the University of California, Davis (TGRC Accession# LA3911). In the present study, we call this the USA line. Micro-Tom was distributed to two research institutes: the Weizmann Institute of Science in Israel and the NARO Institute of Vegetable and Tea Science (NIVTS) in Japan (Fig. 1A). Micro-Tom at the Weizmann Institute was further distributed to the University of São Paulo in Brazil^6^ (BRA line) and to the Institut National de la Recherche Agronomique (INRA) in France^7^ (FRA line). The BRA line is considered genetically similar to the FRA line.^8^ In Japan, Micro-Tom in NIVTS was generated by the single seed descent breeding method. Here, we call this the NIVTS line. The Micro-Tom in NIVTS was distributed to the University of Tsukuba (this line is stored and provided as a part of the National BioResource Project and called the NBRP line) and was further distributed to Kazusa DNA Research Institute (KDRI). The Micro-Tom in KDRI was self-crossed nine times and is called the KDRI line in this study. To identify causal mutations of characteristic traits in Micro-Tom, NBRP line and its mutant lines created by ethyl methanesulfonate (EMS) treatment and gamma irradiation-induced lines have been distributed as bioresources for research communities around the world from the University of Tsukuba.^9,10^

The complete genome sequence of Heinz 1706, a tomato cultivar that has been opened to the public, has been used as a model plant in Solanaceae for breeding and biological research.^11,12^ The genome sequence and its annotation of Heinz 1706 is currently updated to SL4.0 and ITAG4.0^12, 13^ (Hosmani et al., bioRXiv, doi: https://doi.org/10.1101/767764) and has been used as a reference genome of *Solanum lycopersicum*. According to the genome sequence, the cause of dwarfism in Micro-Tom has been clarified by a single-nucleotide polymorphism (SNP) on the 3' end of intron 8 in the Cytochrome P450 gene (Solyc02g089160).^14^ In addition, the cause of self-pruning has been clarified by nonsynonymous SNPs in the SP gene (Solyc06g074350).^15^ Additionally, the cause of uniform ripening, which is absent in dark-green-shouldered varieties or varieties having green patches at the top of the fruit, has been clarified by a single base ‘A’ insertion in the SlGLK2 gene in Micro-Tom.^16^ The polymorphisms that especially influence phenotypes or utilize DNA markers were explored, and genome-wide SNPs between Micro-Tom and Heinz 1706 have been detected by a database survey and a beads array assay.^8,17^ To investigate the difference in the traits of Micro-Tom, the genome-wide SNPs and indels were investigated by mapping short-reads of Micro-Tom S9 (KDRI line) onto the Heinz 1706 genome sequence.^18^ Four EMS-induced mutants and three gamma-ray-irradiated lines of Micro-Tom were also resequenced, and the tendency of mutational substitution of the respective lines has been reported.^19^

Besides, a lot of phenotypic differences within traits such as culm length, number of fruits, and branching among the six lines of Micro-Tom (KDRI, NBRP, NIVTS, USA, FRA, and BRA) were observed (Fig. 1B, Supplementary Data 1). The details of the phenotypes among respective Micro-Tom lines in a greenhouse and a cultivation room are illustrated in Supplementary Data 1 (Fig. SD1-1, SD1-2, SD1-3). Supplementary Data 1 Table 1 summarizes the phenotypic alterations. Overall, four Micro-Tom lines (KDRI, NBRP, FRA, and BRA) are far from the USA line, which is closest to the original Micro-Tom. These four lines exhibit useful phenotypes related to fruit characteristics, such as larger numbers and sizes of fruits. Furthermore, well-curated co-dominant cleaved amplified polymorphic sequence (CAPS) markers^20^ for distinguishing FRA and other lines have been developed (Supplementary Data 1 Table 2), and genotype patterns are illustrated in Supplementary Data 1 (Figures SD1–SD4). According to these results, we supposed that the six Micro-Tom lines might include many polymorphisms. These variations are unique cases originating from a single line and exhibiting diverse phenotypes during propagation through distribution and fixation within a short period of time. Although an analysis using the SNP array of *Solanum lycopersocum* including Micro-Tom^17^ and an analysis by resequencing of KDRI and FRA have been reported,^18^ we considered these analyses insufficient to explore polymorphisms related to their phenotypes because the genome sequence of the Micro-Tom KDRI line was built by mapping that line’s short-reads onto the Heinz 1706 genome sequence (SL2.40). In this study, we conducted a *de novo* assembly of the Micro-Tom KDRI line and built a pseudomolecule. We then compared it with the Heinz 1706 chromosome sequence to reveal structural differences. Furthermore, we reported unique genetic diversities generated across propagations among the six Micro-Tom lines.

## 2. Materials and Methods

### 2.1. Plant materials

We used a Micro-Tom KDRI line distributed from KDRI for the *de novo* genome assembly. This line was derived from Tsukuba University and was self-crossed for nine generations in KDRI^18^ (Fig. 1A). Four other lines, NBRP (TOMJPF00001), NIVTS (AM), FRA (MM), BRA, and USA (LA3911), were obtained from the University of Tsukuba, the NARO Institute of Vegetable and Tea Science, INRA, and the TGRC, respectively.

Since there are some genetic divergences in Micro-Tom,^8,18^ we investigated its distribution path. In 1998, Micro-Tom seeds were transferred from the University of Florida, where it originated,^5^ to NIVTS to establish the NIVTS line. Subsequently, approximately 200–300 seeds of the NIVTS stock were transferred to the University of Tsukuba in 2000, from which NBRP and KDRI were independently established. NBRP was used as a parental line of the EMS mutant libraries^4^ and for BAC library construction.^21^ On the other hand, FRA was established in 2000 (or 2001) from the seed stock of the University of Florida via the Weizmann Institute.^22^ Details of the establishment of LA3911 are unknown.

### 2.2. Whole genome sequencing and assembly of Micro-Tom KDRI line

To reveal the detailed genome structure of Micro-Tom, we have conducted a *de novo* assembly of the Micro-Tom KDRI line by short-reads sequenced by Illumina HiSeq 2000 and MiSeq. We generated a paired-end (PE) library (insert size of 400 bp) with the TruSeq Nano DNA Library Prep Kit (Illumina, San Diego, CA) and five mate-pair (MP) libraries (3 kb, 5 kb, 10 kb, 15 kb, and 20 kb) with the Mate Pair Sample Prep Kit (Illumina). The PCR duplications in Illumina reads were excluded by PRINSEQ^23^ (v0.20.4). The remaining reads were trimmed with quality scores < 10 by PRINSEQ. The genome size of the Micro-Tom KDRI line was estimated using GenomeScope 2.0^24^ with Illumina MiSeq PE reads. The adaptor sequences in the reads were trimmed by fastx_clipper in the FASTX-toolkit (http://hannonlab.cshl.edu/fastx_toolkit/). The assembly procedure is summarized in Supplementary Figure S1. The PE reads of Illumina MiSeq and the MP reads of HiSeq 2000 were assembled by MaSuRCA (v2.3.2).^25^ To exclude the probable contaminated sequences, the scaffolds were searched against the NCBI bacterial and fungal genomes, UniVec, human genome (hg19), and the chloroplast genomes of *Solanum lycopersicum* (Accession: NC_007898), *S. tuberosum* (NC_008096.2), *S. bulbocastanum* (NC_007943.1), *Arabidopsis thaliana* (NC_000932.1), the mitochondrial genome of *S. lycopersicum* (SOLYC_MT_v1.50; https://www.ncbi.nlm.nih.gov/nuccore/AFYB00000000.1/), *Nicotiana tabacum* (NC_006581), *A. thaliana* (NC_001284), and PhiX used for Illumina sequencing by BLAST^26^ with an E-value cut-off of 1E–10. The remaining scaffolds were further connected with BAC end sequences obtained in a previous study^21^ by using SSPACE 2.0.^27^ Next, HiSeq PE reads (DRR000741) at a coverage of 68.9-fold and MiSeq PE reads used in genome assembly were employed to close the gaps by using GapFiller.^28^ Furthermore, the super-scaffolds were aligned to the Heinz 1706 (SL4.0) genome by RaGOO v1.1.^29^

### 2.3. Comparison of the genome sequences of KDRI and Heinz 1706

Two chromosome sequences, SLM_r1.2 and SL4.0, were compared with NUCmer^30^ and the polymorphisms (SNPs and indels) were detected with the show-snps function of MUMMER (v3.23).^30^ The alignments of the chromosome sequences between SLM_r1.2 and SL4.0 and between the two lines of Micro-Tom, SLM_r1.2, and FRA (SLYMIC: GenBank accession: CM022782.1 to CM022793.1) were represented using MiniMap2 (v2.24).^31^ Full-length cDNA sequences of Micro-Tom^32^ (DDBJ accession numbers AB211519-211522, AB211526, AK224591-AK224910, AK246135-AK248077, and AK319176-330134) were mapped to SLM_r1.2 using sim4db version r1896.^33^

### 2.4. Gene prediction and annotation of Micro-Tom KDRI

Gene prediction of Micro-Tom KDRI line was performed by an in-house pipeline^34^ (Supplementary Fig. S2). The initial step of the pipeline involved predicting genes *ab initio* against SLM_r1.2 using BRAKER2 (v2.1.0).^35^ This was done utilizing RNA-Seq reads from Micro-Tom (DRA accession SRA10152) and the amino acid sequence of Heinz 1706 (ITAG4.0). Sequence similarity searches were conducted against UniProtKB^36^ and NCBI’s nr proteins^37^ using DIAMOND (version 0.9.30.131)^38^ in the ‘more-sensitive’ option, employing a filter of *E*-value ≤ 1e–20 and identity ≥25%. Next, gene expression analysis was performed by Salmon (v1.2.1),^39^ and the genes with TPM values > 0.1 were considered expressed. The protein domains of the genes were searched by HMMER (v3.2.1)^40^ against Pfam31.0^41^ with an *E*-value of 1e–30. The genes were queried against ITAG4.0 and Araport11 peptide sequences^42^ using BLAST^26^ with specific criteria: an *E*-value of 1e–20 and a similarity threshold >65% along with a length coverage exceeding 50% for ITAG4.0,^12^ and an *E*-value of 1e–20, a similarity of ≥40%, and length coverage > 50% for Araport11. As performed in the previous study,^34^ genes whose product names, identified through the UniProtKB^36^ search, contained keywords related to transposable elements (TE) were categorized as TE genes. The genes that retained sequence similarity hits and were also found to be expressed by Salmon^39^ were designated as high-confidence (HC) genes, while the remaining genes were categorized as low-confidence genes.

### 2.5. Resequencing of the six Micro-Tom lines

The polymorphism analysis was performed by using the whole genome resequencing data for the six lines of Micro-Tom (KDRI, NBRP, NIVTS, FRA, BRA, and USA). The resequencing data of the NBRP and FRA lines were obtained from the NCBI SRA database (accession numbers: DRR000741 and ERR340383). Resequencing data of the NIVTS and BRA lines were also obtained. The sequencing method was as follows. Genome libraries were constructed with the TruSeq DNA PCR-Free Sample Prep Kit (Illumina) in accordance with the manufacturer’s protocols. The libraries were sequenced by Illumina HiSeq 2000 in 251 bp PE and Illumina NextSeq 500 in 76 bp PE mode. The obtained sequence reads were trimmed with PRINSEQ^23^ and fastx_clipper in FASTX-toolkit (http://hannonlab.cshl.edu/fastx_toolkit).

### 2.6. Genotyping of the six Micro-Tom lines

SLM_r1.2 was used as a reference and softmasked by RepeatMasker (v1.332) (http://www.repeatmasker.org) with the ‘–xsmall’ option. The masked nucleotides were converted to ‘n’ by an in-house Perl script. SNP detection was performed using the pipeline provided from Plant GARDEN.^43^ The trimmed reads were mapped onto SLM_r1.2 with Bowtie2 (v2.3.5.1),^44^ and SNP calling was performed using Samtools (v1.9)^45^ and Bcftools (v1.9).^46^ Homozygous SNPs between the mapping result of KDRI and SLM_r1.2 were filtered based on the following criteria: the genotype represented ‘0/0’, a minimum quality value greater than 999, and a minimum depth of more than 8. To illustrate graphical genotypes among the six Micro-Tom lines, SNPs were chosen at intervals of approximately 500 kb on the chromosomes. A graphical genotyping table was made with Microsoft Excel VBA. Nonsynonymous SNPs and indels, which could be candidates to influence phenotypes, were curated by SNPeff v4.3t^47^ using the SL4.0 Heinz genome as a reference. Additionally, the trimmed reads from the KDRI line were aligned to the genome sequence of the Micro-Tom FRA line (Micro-Tom MM) named SLYMIC with Bowtie2.^44^ SNPs and small indels were searched with mpileup in Samtools^45^ and filtered with Vcftools.^48^

## 3. Results and discussion

### 3.1. Construction of pseudomolecule of KDRI line, SLM_r1.2

**Figure 2.**
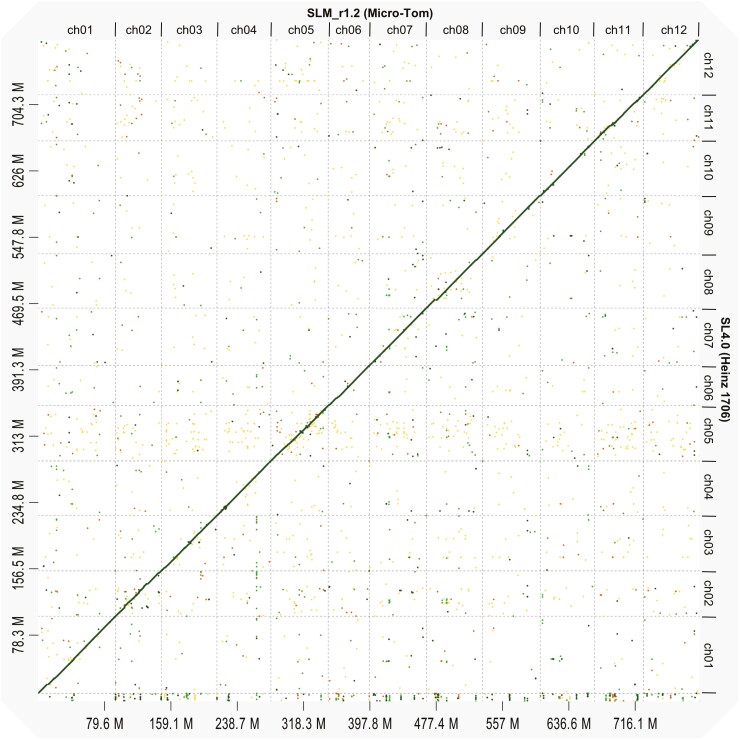
Genome alignment between SLM_r1.2 (Micro-Tom KDRI line) and SL4.0 (Heinz 1706). The horizontal axis indicates the chromosome numbers (ch01 to ch12) of the SLM_r1.2 (KDRI line). The vertical axis indicates SL4.0. The genome alignments in each chromosome are shown in Supplementary Figure 5.

The combined coverage of the trimmed reads of 69M PE and 269M MP was estimated to be 61.4-fold that of the Micro-Tom genome (Supplementary Table S1). The trimmed PE and MP reads were assembled into 48,634 contigs and 16,580 scaffolds, of which 8,008 were excluded for probable contamination. The remaining 8,572 scaffolds were further connected with BAC end sequences, and then 8,522 super-scaffolds were constructed. The HiSeq PE reads (DRR000741) and MiSeq PE reads were used to close the gaps, and 2,925 super-scaffolds with a length of ≥2 kb were selected as the draft sequence, which we named SLM_r1.1. The super-scaffolds were then aligned to the Heinz 1706 (SL4.0) genome, and the resultant sequences were designated SLM_r1.2. The statistics of the sequences obtained at each step are summarized in Supplementary Table S2. SLM_r1.2 comprised 12 chromosomes, excluding chr00, which was the same as in other *Solanum lycopersicum* families.^8^ The combined length of ch01 to 12 was 793,938,277 bp, and when including chromosome 00, the total length was 795,697,831 bp. In addition, it was obvious that the length of ch00 was made up of 55.7% Ns, 0.8% repetitive sequences, comprising 0.45% simple repeats and 0.35% low complexity sequences, with no encoded DNA/RNA transposons. The sequence alignments from ch01 to ch12 between SLM_r1.2 and SL4.0 displayed a diagonal line, indicating the absence of large translocations. We confirmed that there were no critical mis-assemblies in SLM_r1.2 (Fig. 2). The estimated genome size of Micro-Tom KDRI line, calculated from the *k*-mer frequency plot with a *k*-mer size of 21, was 775,851,279 bp. The statistics of the pseudomolecules of the SLM_r1.2 (KDRI line), SLYMIC (FRA line), and ITAG4.0 (Heinz 1706) are summarized in Supplementary Table S2. We considered the assembled genome size of SLM_r1.2 to correspond to the estimated size (Supplementary Figure S3). On the other hand, the total length of Heinz 1706 from ch01 to ch12 was 772,876,783 bp, and the total length was 782,520,033 bp including 9,643,250 bp of ch00. A comparison of the genome sequences of SL4.0 and SLM_r1.2 showed that the latter was slightly longer than the former, by approximately 13.2 Mb. In addition, the total length of the chromosomes (chr01-chr12) of Micro-Tom FRA line (SLYMIC) was 791,327,130 bp. The total length was also slightly longer than that of SL4.0, by 18,450,347 bp. The genome sequence SLM_r1.2, spanning from ch01 to ch12, was found to encode 97.0% of the universal single-copy genes from the embryophyta_odb10 dataset in BUSCO software.^49^ Notably, these genes were not detected on ch00 (Supplementary Table S4). SLM_r1.2 is noted for its high-accuracy gene content without any excess or deficiency. Out of the 13,227 full-length cDNA sequences in Micro-Tom,^32^ 12,817 (96.9%) were successfully mapped to SLM_r1.2. The 410 unmapped sequences corresponded to 1,725 redundant or chimeric sequences.^32^ According to these results, the quality of the genome sequence of SLM_r1.2 could be considered sufficiently high for application to further analyses.

### 3.2. Distribution of polymorphisms between SLM_r1.2 and SL4.0

Table 1 summarizes the total lengths and polymorphisms found in SLM_r1.2. We detected 804,351 SNPs, 117,981 insertions, and 79,359 deletions between SLM_r1.2 and SL4.0 by using Nucmer (Supplementary Data 2). In a previous study, genetic distances between Micro-Tom and Heinz 1706 indicated that these Micro-Tom lines could be divided into different clusters on a dendrogram through genotyping using array assays with 7,054 SNPs.^17^ One of those clusters was composed of five inbred Micro-Tom lines, while the other was composed of Heinz 1706 with 15 inbred lines and two lines of *S. lycopersicum* var. cerasiforme. Those clusters were found to include a cluster composed of five F_1_ hybrid cultivars and an inbred line. In another previous study, genome-wide polymorphisms detected between the re-sequenced Micro-Tom (hereafter SLM) and Heinz 1706 (SL2.40) were estimated to be 1,231,191 SNPs and 117,354 indels.^18^ Fewer SNPs were detected in SLM_r1.2 than in SLM, indicating that the sequence quality was increased by *de novo* assembly of Micro-Tom and the update of Heinz 1706 (SL2.40 to SL4.0). Furthermore, the distribution of SNPs in each chromosome was similar between the SLM reported previously^18^ and SLM_r1.2. The distributions of SNPs in SLM_r1.2 in ch01, ch03, and ch07 were especially very similar to each other (Supplementary Fig. S4A). The positions of the peaks, including high numbers of SNPs, were dispersed across each chromosome. Similar tendency of SNPs’ distribution was observed among Japanese rice cultivars developed around the time of World War II and later.^50^ The cause was seemed that those genomes included differences between genetically similar regions inherited by close relatives and relatively distant regions. It was also reported changes in crop diversity termed crop genetic erosion, which are significantly reduced just before the diversity expansion brought about by the Green Revolution in the 1960s, in subsequent years, loss of allelic diversity in chromosome segments, and uniformity in cultivated crops.^51^ Similar situations arose during tomato domestication.^2,52,53^ The bias in SNP distribution between SLM_r1.2 and SL4.0 in the tomato genome was influenced by the rapid diversification of closely related cultivars. Although the total numbers of indels in SLM_r1.2 (117,981 insertions and 79,359 deletions) were slightly higher than those of indels in SLM (111,354 insertions and 70,124 deletions),^18^ the total length of indels in SLM_r1.2 (341,732 bp in insertion and 302,459 bp in deletion) was longer than that in SLM (272,863 bp in insertion and 177,311 bp in deletion) (Table 1). Although the average length of insertions (2.9 bp) was shorter than that of deletions (3.8 bp) (Supplementary Table S5), the number of insertions (341,732) was more than that of deletions (79,359) in SLM_r1.2. As a result, the total length of insertions was longer than that of deletions, which could be considered as one reason why the total length of SLM_r1.2 was longer than that of SL4.0. The distributions of indels and SNPs show a positive correlation (Supplementary Table S5), consistent with previous reports on structural variations including indels and SNPs discussed in the context of linkage disequilibrium.^54–56^ Furthermore, we detected SNP distribution between SLM_r1.2 and Micro-Tom FRA (Supplementary Fig. S4B). The positions and biases of the SNPs between the two Micro-tom lines were similar to those between Micro-Tom and Heinz 1706. On the other hand, some peaks indicating high numbers of heterozygous SNPs were observed within the Micro-Tom genome (Supplementary Fig. S4B). Although some of the crowded heterozygous SNPs were associated with loci that represented peaks of SNPs between KDRI and FRA lines, such as 9–14 Mb on ch08 and 18–19 Mb and 50–52 Mb on ch11. In these regions, homozygous SNPs were mostly present in the USA line, whereas heterozygous SNPs were present at many positions in the NBRP, NIVTS, FRA, and BRA lines. In the KDRI line, most of the heterozygous SNPs were located apart from the SNPs between the close lines. These regions including a large number of heterozygous SNPs may suggest to be still genetically unstable regions.

**Table 1.** Summary of polymorphisms between the genomic sequences of Micro-Tom S9 (KDRI line) (SLM_r1.2) and Heinz 1706 (SL4.0)

Chrs	Length of chromosomes (bp)	Number of SNPs	Number of insertions	Length of insertions (bp)	Number of deletions	Length of deletions (bp)
ch01	92,770,581	39,646	12,542	28482	7,290	26,333
ch02	54,804,935	88,607	9,296	27,869	6,044	23,662
ch03	66,762,231	68,767	9,513	31,596	7,448	32,006
ch04	64,756,891	84,031	15,473	45,850	11,515	42,324
ch05	68,728,436	243,847	19,606	58,382	13,012	44,148
ch06	49,296,944	10,691	5,303	9,368	3,003	10,698
ch07	68,511,004	84,776	13,240	46,962	10,524	41,813
ch08	67,510,114	13,649	5,129	11,337	2,626	10,241
ch09	69,060,779	27,424	7,432	19,758	4,375	16,467
ch10	65,174,262	10,179	5,335	10,536	2,367	9,746
ch11	58,512,243	105,007	9,746	36,092	7,562	29,537
ch12	68,049,857	27,727	5,366	15,500	3,593	15,484
ch00	1,759,554	–	–	–	–	–
Total	795,697,831 (793,938,277;except chr00)	804,351	117,981	341,732	79,359	302,459

### 3.3. Gene annotation of SLM_r1.2

According to the gene prediction, 34,526 HC genes were predicted in SLM_r1.2 (27 of which were in ch00) (Supplementary Table S4, Supplementary Data 3, and Supplementary Data 4). Of those 34,526 CDSs, 34,499 (99.92%) had both start and stop codons (13927: ATG and TGA, 11636: ATG and TAA, 8936 ATG and TAG). On the other hand, 19,157 (85.57%) of 34,075 CDSs had both start and stop codons (11498: ATG and TGA, 10575: ATG and TAA, 7084 ATG and TAG) in ITAG4.0. According to these results, most of the predicted genes in SLM_r1.2 were identified as complete CDSs compared to those in ITAG4.0. The genes in chromosomes from ch01 to ch12 in SLM_r1.2 were compared with those in ITAG4.0. Of the 34,499 genes in SLM_r1.2 (Micro-Tom), 24,136 (70.0%) genes corresponded to 29,222 (87.0%) of the 33,562 genes in ITAG4.0 (Heinz 1706) (Supplementary Fig. S5). The number of predicted genes in SLM_r1.2 is greater than that in SL4.0, and the genome size of SLM_r1.2 is longer than that in SL4.0. In the aligned regions between SLM_r1.2 and SL4.0, the gene densities were 23,013 and 23,028 bp/gene, respectively. Moreover, 368 genes were found only in SLM_r1.2 genomic regions, while 224 genes were not identified in SL4.0 (Supplementary Table S6). From these comparisons, it did not appear that the predicted genes in SLM_r1.2 significantly outnumbered those in SL4.0.

### 3.5. Genome sequence comparison between SLM_r1.2 and SLYMIC of French Micro-Tom

Two Micro-Tom lines, FRA and KDRI, were each compared by mapping the short-reads against the reference genome of Heinz 1706 (SL2.40).^18^ The complete genome sequence of the FRA line (SLYMIC) has been successfully assembled using a combination of PacBio sequencing, Illumina HiSeq sequencing, and BioNano optical mapping techniques. The genome structures of SLM_r1.2 and SLYMIC (Supplementary Figs S6A2-M2) were very similar, but some regions were different. For example, there were insertions of SLM_r1.2 to SLYMIC in 10 Mb of Ch06 and 10 Mb of Ch08 (Supplementary Figs S6G2 and S6I2). Especially, the deletion observed in the assembled genome sequence of Ch06 was thought to be SLYMIC-specific, because 10 Mb of SL4.0 was filled (Supplementary Fig. S6G1). Short deletions of 35 Mb and 40 Mb were detected on chromosome 12 of SLM_r1.2 but were not found in the SLYMIC genome. These differences in length between insertions and deletions were related to differences in total length between Micro-Tom lines. Furthermore, there was a fragment in the complementary direction at 33 Mb in Ch03 (Supplementary Fig. S6D2). However, there was no fragment in the alignment between SLM_r1.2 vs. SL4.0 (Supplementary Fig. S6D1). In this case, we evaluated the accuracy of SLYMIC, considering that it was constructed using long-reads and optical mapping, while SLM_r1.2 was based on short-reads.

### 3.6. Polymorphism analyses among the six Micro-Tom lines

**Figure 3.**
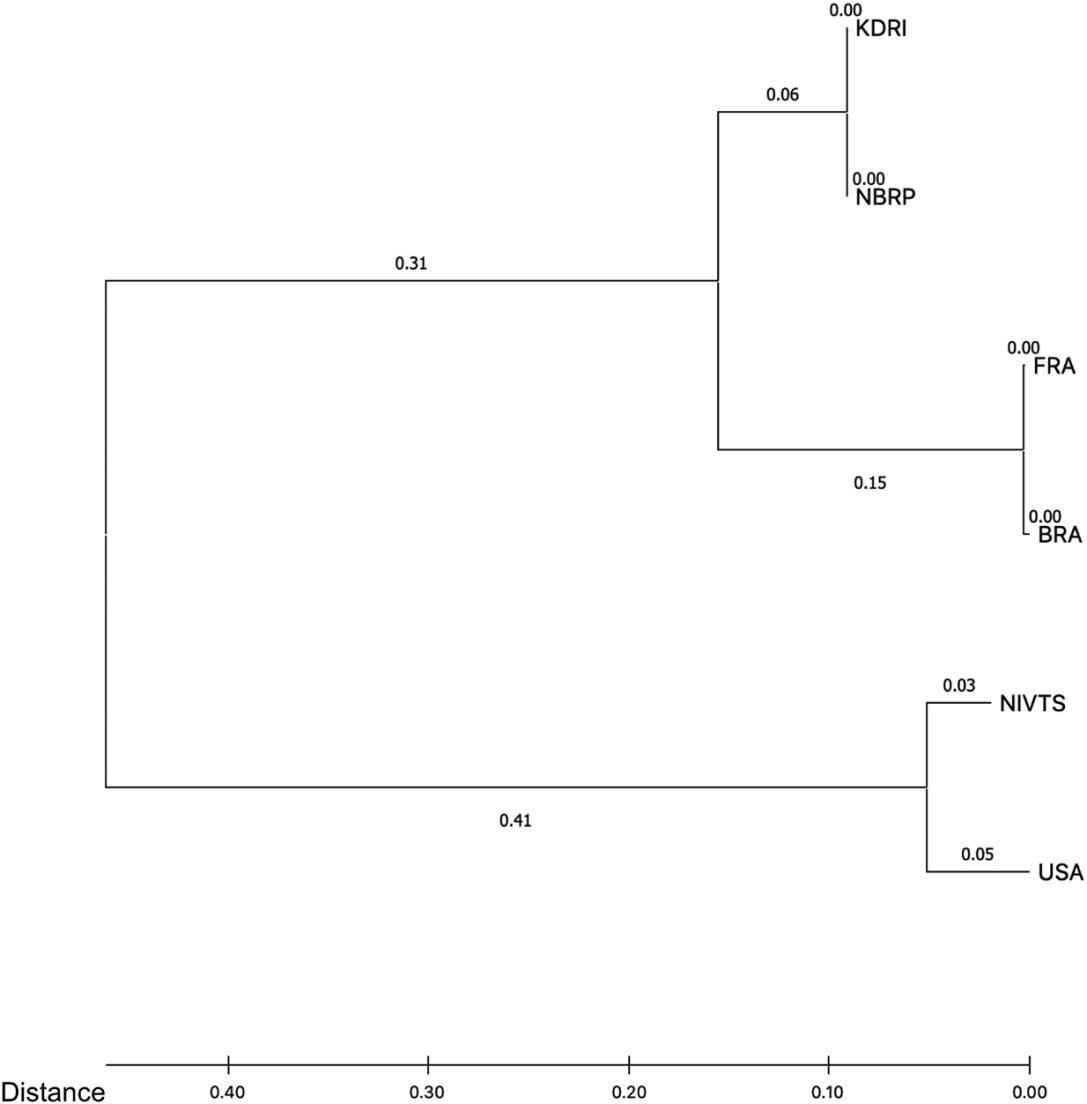
A dendrogram among the six Micro-Tom lines using 325,361 SNPs, which are distributed across the six Micro-Tom lines. KDRI; Distributed from Kazusa DNA Research Institute in Japan, NBRP; Distributed from University of Tsukuba in Japan, NIVTS; Distributed from NARO Institute of Vegetable and Tea Science in Japan, FRA; Distributed from INRA in France, BRA; Distrubuted from USP in Brazil, USA; Distributed from TGRC at UC Davis in USA. The values indicate the branch lengths.

The total number of polymorphisms found among the six Micro-Tom lines were 325,361 SNPs and 9,084 indels. Using these SNP data, a dendrogram by the neighbour-joining method was formed (Fig. 3). FRA and BRA lines were positioned close to each other in the dendrogram, indicating their similarity. These lines also exhibited similar phenotypes, such as long internodes in cultivation room environments and larger fruits in greenhouse environments. Similarly, NBRP and KDRI lines were closely positioned in the dendrogram, and these lines exhibited a high fruiting rate as well as similar plant postures in cultivation room environments and greenhouse environments (Supplementary Data 1 Table 2). A dendrogram among tomato wild types and mutant lines, including the six Micro-Tom lines and was based on 1,112 mutations (using well-curated SNP and indel candidates), was previously reported.^19^ In comparison to these dendrograms, the USA and NIVTS lines are close in the new tree (Fig. 3), which corresponds to the propagation path of Micro-Tom lines (Fig. 1A). However, the pair of KDRI and NBRP, as well as that of FRA and BRA, are clustered closely together, unlike the propagation pattern, and they are distant from the pair of NIVTS and USA. On the other hand, the phylogenetic tree constructed by Shirasawa et al.^19^ illustrated the distances of the pairs between USA and NIVTS lines, KDRI and NBRP lines, FRA and BRA lines were close, whose branching pattern was consisted with that constructed in this study. According to the phylogenetic tree constructed in this study, the mutations were accumulated in different manners between the USA and NIVTS lines and the remaining lines, and those also were accumulated in different manners between the NBRP and KDRI lines, and between FRA and BRA lines. This may mean that unfixed genetic variations were fixed separately in each pair of lines after the propagation.

### 3.7. Characteristics of genotype patterns among six Micro-Tom lines

**Figure 4.**
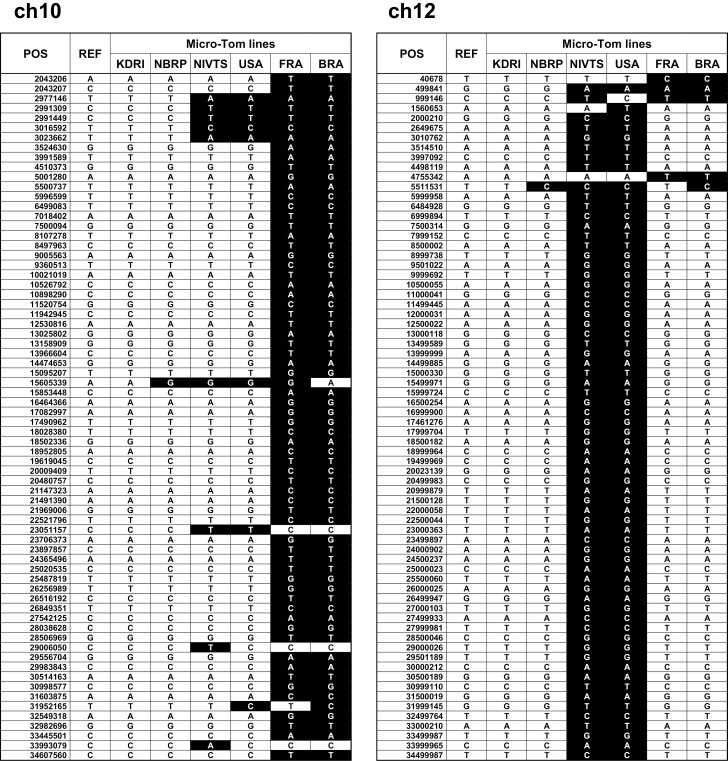
The regions containing SNPs in ch10 and ch12 of six Micro-Tom lines. The genomic sequences of six Micro-Tom lines were compared individually with the reference sequences termed SLM_r1.2 for chromosomes 10 and 12. The SNP positions (POS), the reference sequence at that position (REF), and the corresponding sequences in KDRI, NBRP, NIVTS, USA, FRA, and BRA lines are shown, with the sequences that differ from the reference indicated in white under black background. As it is difficult to show the entire chromosomes, only a portion of chr10 from 2043206 to 34607560 (total length: 65174262 bases), and another of chr12 from 40678 to 34499987 (total length: 68049857 bases) are shown. The sequences of KDRI and NBRP, NIVTS and USA, and FRA and BRA lines, respectively, are similar to each other, which may also be seen in the dendrogram shown in Fig. 3. The whole reference sequence of SLM_r1.2 is presented in Supplementary Data 2.

To evaluate the differences in the genomes of the six Micro-Tom lines throughout their propagation and distribution, we conducted genotype comparisons among these lines (Supplementary Data 5) and subsequently constructed graphical representations of their genotypes (Supplementary Data 6). The genotyping patterns of the six lines were mosaic-like and chimeric throughout the chromosomes, except for ch10 and ch12. On the other hand, genotype patterns represented large blocks in whole chromosomes of ch10 and ch12 (Fig. 4). The genotype patterns of ch10 and ch12 were separated into three groups. The first was NBRP and KDRI, the second was NIVTS and USA, the other was FRA and BRA. In the case of ch10, the genotype pattern of KDRI and NBRP appeared to be similar to the pattern observed in NIVTS and USA, whereas the genotype pattern of FRA and BRA, which were close in the propagation process, showed significant differences from these patterns. These observed patterns seemed to correspond to the propagation path. It is interesting and inconsistent with the propagation process that in ch12, the genotype patterns across the lines of NIVTS and USA, which were generated earlier, appeared similar to the patterns of the FRA and BRA lines, as well as those of the NBRP and KDRI lines, which were on the distant ends of the propagation process, indicating potential common genotypes. We found the genetic variations in Micro-Tom lines may have been insufficient fixation of the original Micro-Tom line, especially we found the large blocks including different genotype patterns in ch10 and ch12. We consider that the genetic variations have been already clustered together in the original Micro-Tom, and these variations became apparent as genetic fixation progressed. It has been reported that there are resistance gene loci against various fungi and viruses, such as *Phytophthora infestans*,^57^ Tomato yellow leaf curl virus, and Tomato mottle virus^58^ around ch10. On ch12, the loci related to fruit traits, which were fruit morphology,^59^ cuticle,^60^ and lycopene content,^61^ were reported using recombinant isogenic lines of tomatoes. It is supposed that these loci are influenced by the distinct genotypes and disease-resistance characteristics of each line. However, these genotype patterns seem unique and represent the genetic fixation process.

### 3.8. Relationship between phenotypes and polymorphisms among six Micro-Tom lines

Among various tomato cultivars, including Micro-Tom, it has been previously reported that certain alleles of SNPs and indels have an impact on phenotypic traits.^14–16,62–64^ There are various phenotypes among the six Micro-Tom lines (Supplementary Data 1), such as glossy fruit, shoot branching, leaf serration, and bearing/ripening. Among the predicted genes in SLM_r1.2, 1,963 were identified as having nonsynonymous mutations that affect the amino acid sequences of their transcripts. These mutations were annotated as 6,148 missense variants and 8 stop-retained variants using SNP annotation with SnpEff (Supplementary Data 7). We conducted a survey to investigate the presence of alleles via SNPs and indels in 29 genes that are potentially influential in determining phenotypic traits among different tomato cultivars^12,65–70^ (Supplementary Data 8). This revealed that no candidates represented genotypes corresponding to phenotypes among the six Micro-Tom lines. However, the possible causes are not difficult to explain. First, the tomato cultivars in which the mutation was reported were not Micro-Tom. If the cultivars containing the mutation are not genetically closely related to Micro-Tom, it is possible that the causative mutation of the gene is different. The second point to consider is that these phenotypes are inferred from Quantitative Trait Loci, which are known to be associated with many polygenes.^71,72^ The third is epigenetic regulation of gene expression and changes in traits.^73^ These results suggest that Micro-Tom lines contain cryptic mutations and genetic functions. Therefore, the identification of causative genes for these phenotypes should be pursued through further investigations. The diversification of crops has been reported to occur during clonal propagation and domestication processes.^74,75^ While the propagation method of the Micro-Tom lines involves seed propagation, this can result in genetic variations and the segregation of traits, especially when genetic fixation is incomplete or when artificial selection is applied. It is noteworthy that artificial selection can be both intended and unintended. It is possible that while growing in an artificial environment, that is, cultivation room or greenhouse, selection may have unintentionally been applied for the plant to adapt to such an environment. The presence of a variety of phenotypes in the Micro-Tom is seen as an advantage in its role as a model plant.

### 3.9. Crucial data for effective bioresource management of Solanaceae family

The usefulness of Micro-Tom as a model plant^4,9,10^ and its genetic distance from Heinz 1706, the reference genome of the Solanaceae family,^12^ have been investigated previously.^17–19^ Although resequencing of the KDRI line has been reported, the genome sequence was constructed through mapping-based methods.^18^ In this study, the KDRI genome sequence was assembled *de novo* and a chromosome-scale pseudomolecule was constructed by referring to the genome sequence of Heinz 1706. The genome sequence of Micro-Tom, determined through *de novo* assembly, has been made publicly available for the first time in this study. Additionally, genetic comparisons between Micro-Tom and Heinz 1706 have been updated from previous research conducted in SLM.^18^ We revealed the genetic diversity among the six Micro-Tom lines and found that Micro-Tom’s diversity is unique because it is formed among close (originally thought to be the same) lines and includes characteristic genotypes on ch10 and ch12. Although the alleles that directly affect the phenotypes among the various six lines were not clearly identified, Micro-Tom has been distributed worldwide from six organizations and is used as a model plant. Currently, the genome sequences at the chromosome-level have been determined in many organisms by using long-reads such as HiFi reads obtained by Sequel2/Revio (Pacific Biosciences, CA, USA) or Nanopore reads obtained by MinION/GridION/PromethION (Oxford Nanopore Technologies, Oxford, UK). In this study, we have determined the genome sequence of Micro-Tom S9 (KDRI) for assembly using short-read data. We consider that the assembled sequence (SLM_r1.2) in this study is of high quality, because the BUSCO completeness was 97.0% (Supplementary Table S3). In this study, the genetic differentiation of the phenotypes and genotypes in the Micro-Tom genome sequence occurred by genetic fixation during a short period of time propagation. It is highly possible that the original line had internal genetic variations that have become apparent in later generations. If we obtain HiFi reads of the six Micro-Tom lines, we can construct phased genome sequences by haplotype-resolved *de novo* genome assembly and can identify the regions with unfixed genetic variations and find the relationship between the phenotypes and genotypes among the Micro-Tome lines. The implications of these findings are substantial, which include various phenotype information such as fruit related and CAPs markers (Supplementary Data 1, Supplementary Data 1Table 1 and 2) and genotypes of the six Micro-Tom lines will be crucial data for effective bioresource management.

## Supplementary Material

dsae016_suppl_Supplementary_Figures

dsae016_suppl_Supplementary_Tables

dsae016_suppl_Supplementary_Data_S1

dsae016_suppl_Supplementary_Data_S1

dsae016_suppl_Supplementary_Data_S2

dsae016_suppl_Supplementary_Data_S3

dsae016_suppl_Supplementary_Data_S4

dsae016_suppl_Supplementary_Data_S5

dsae016_suppl_Supplementary_Data_S6

dsae016_suppl_Supplementary_Data_S7

dsae016_suppl_Supplementary_Data_S8

## Data Availability

All the sequences described in this paper are available from the DNA Data Bank of Japan (DDBJ) with accession numbers. The accession numbers for the SLM_r1.2 pseudomolecule of the Micro-Tom KDRI line are BSVZ01000001-BSVZ01000165. The sequence reads of the KDRI line used for genome assembly and mapping are DRR118528-DRR118529. The sequence reads used for resequencing of other Micro-Tom lines are DRR022704 for NBRP; DRR441010 for NIVTS; DRR118570-118571 for USA; ERR340383-340384 for FRA; and DRR441011 for BRA. Supplementary data, figures, and tables are available from FigShare (https://figshare.com/s/c687430507701cca5ae1).
