# Supplementary material for: Genomic variation across distribution of Micro-Tom, a model cultivar of tomato (Solanum lycopersicum)
Source: DNA Res. 2024 Jun 7;31(5):dsae016. doi: 10.1093/dnares/dsae016 (PMC11481021; doi:10.1093/dnares/dsae016)

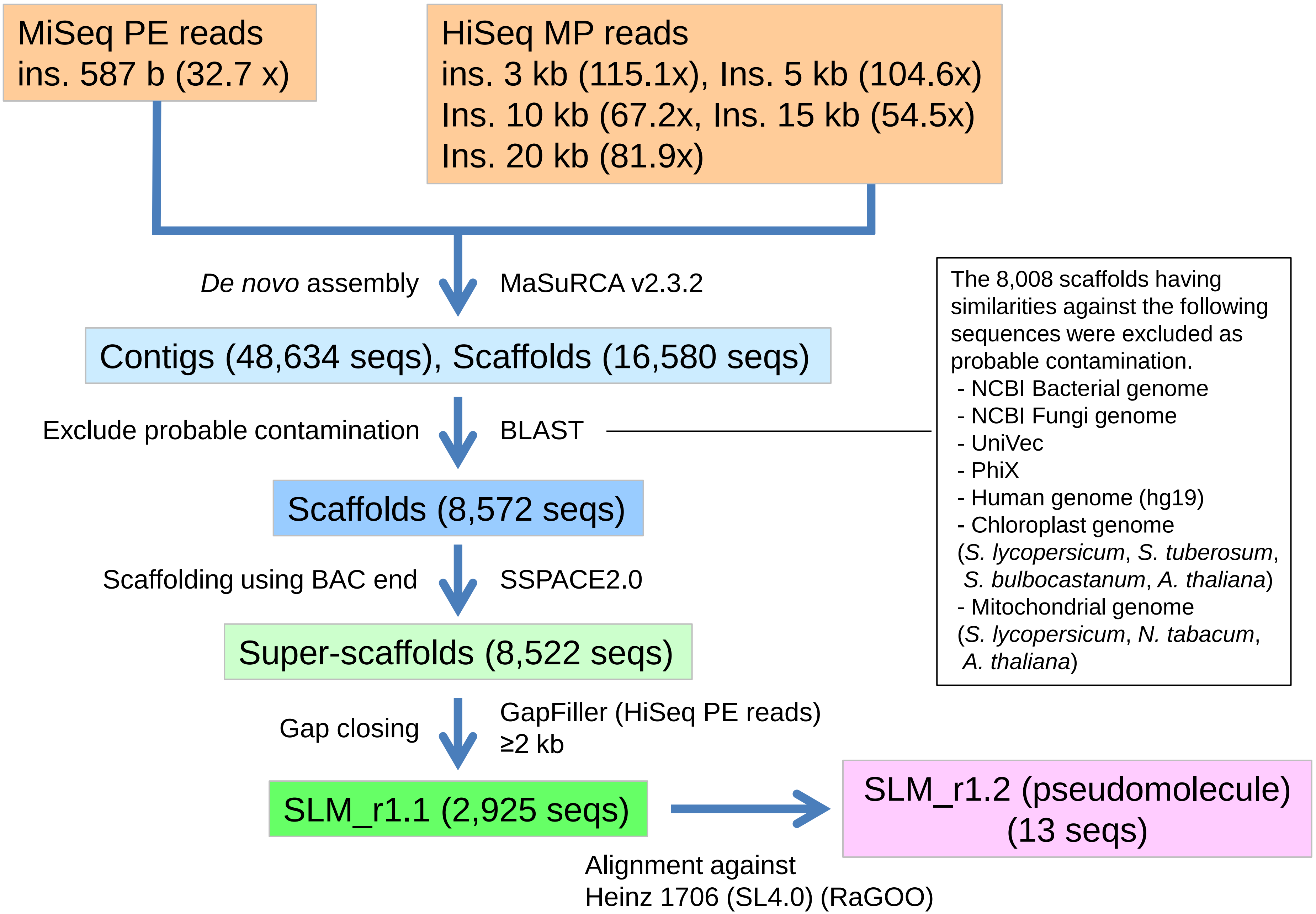

Supplementary Figure S1. Procedure of genome assembly for Micro-Tom S9

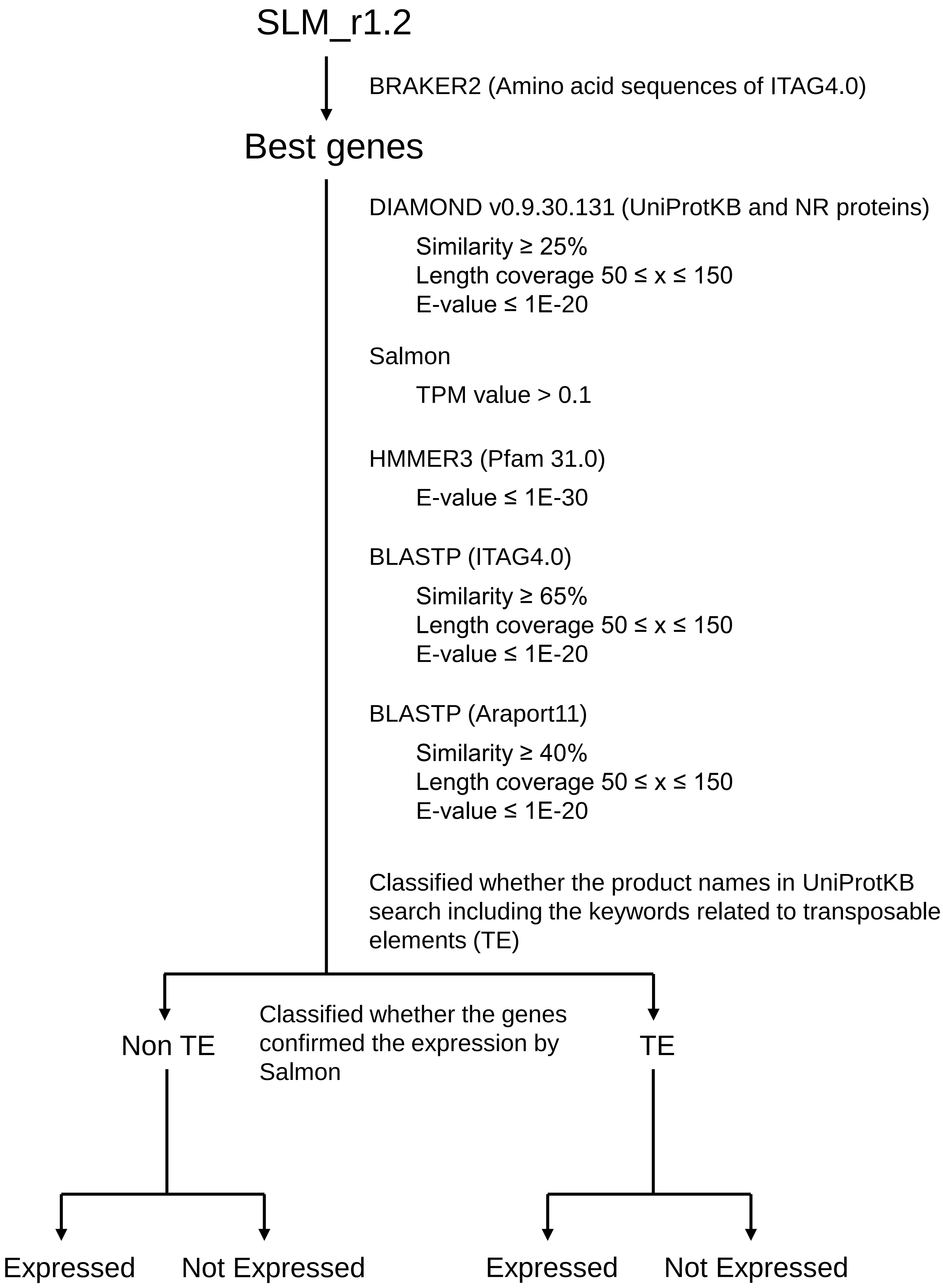

Supplementary Figure S2. Procedure of in-house gene prediction

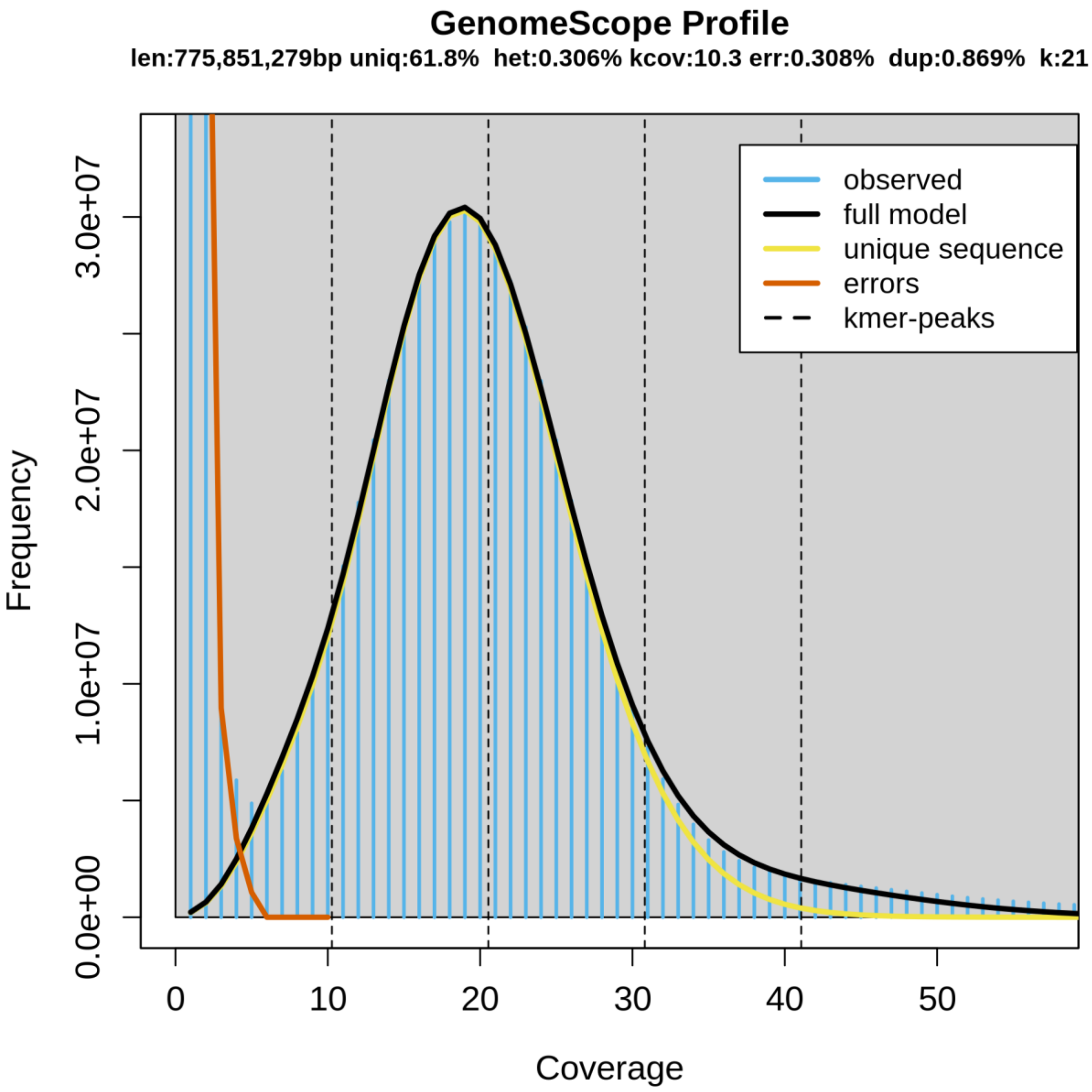

Supplementary Figure S3. *K*-mer frequency distribution of Micro-Tom KDRI line.
Genome size of Micro-Tom KDRI line was estimated using GenomeScope 2.0 with Illumina MiSeq reads in *k*-mer size 21.

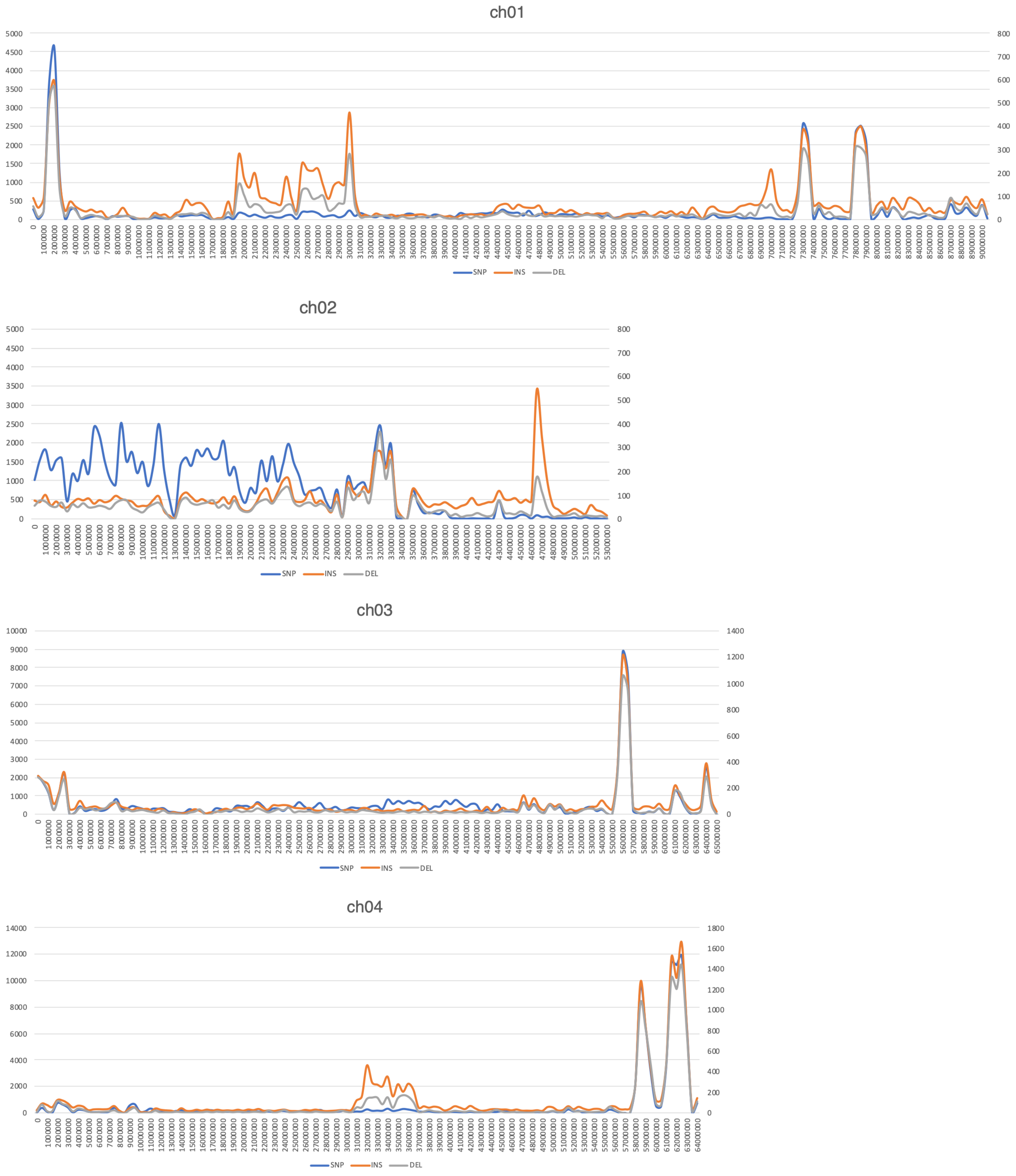

Supplementary Figure S4a. Distributions of polymorphisms between Micro-Tom S9 (KDRI line) and Heinz 1706 in tomato 12 chromosomes

The horizontal line represents the physical distance along respective chromosome, split into 500 kb windows. The right vertical line indicates the number of SNPs for blue line indicates SNPs. The right vertical line indicates the number of indels. The orange line indicates insertions and the gray line indicates deletions, respectively.

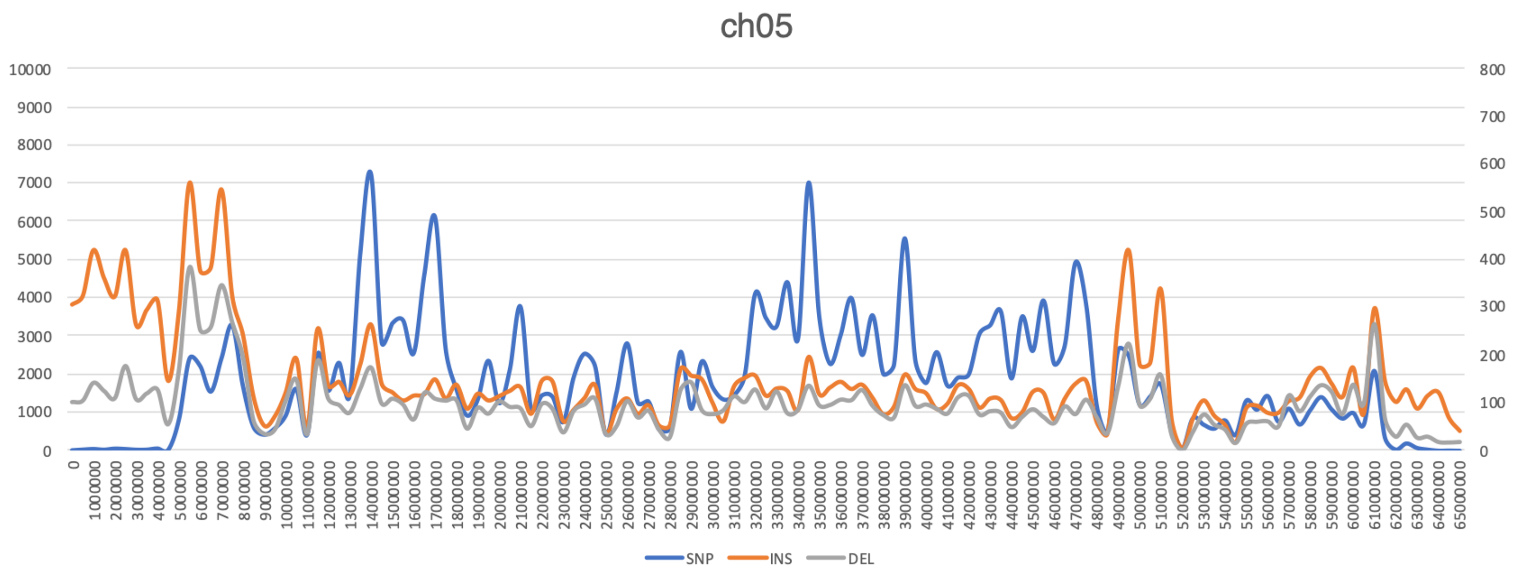

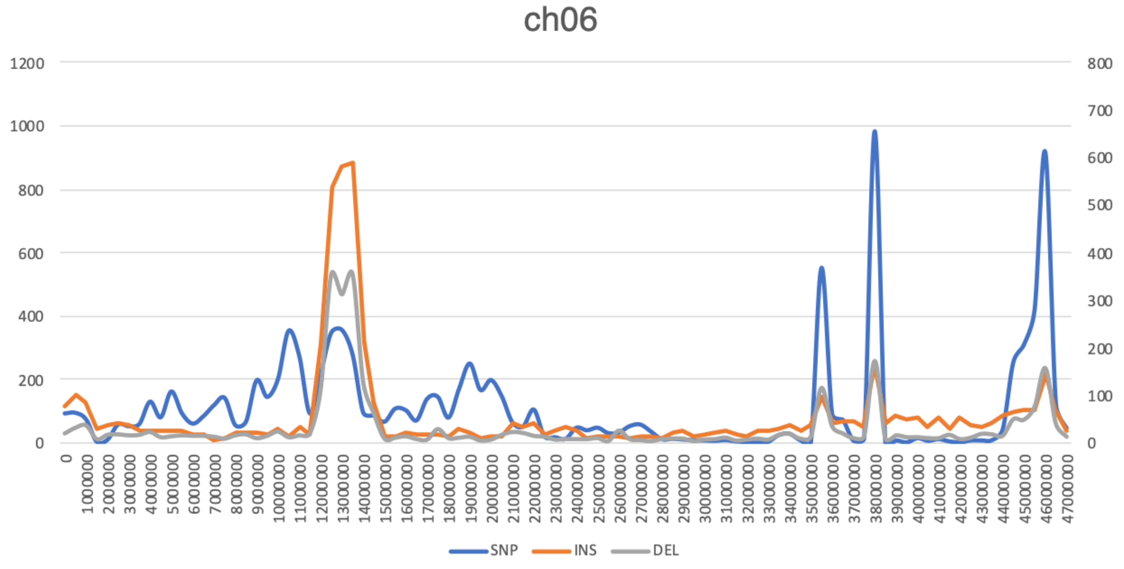

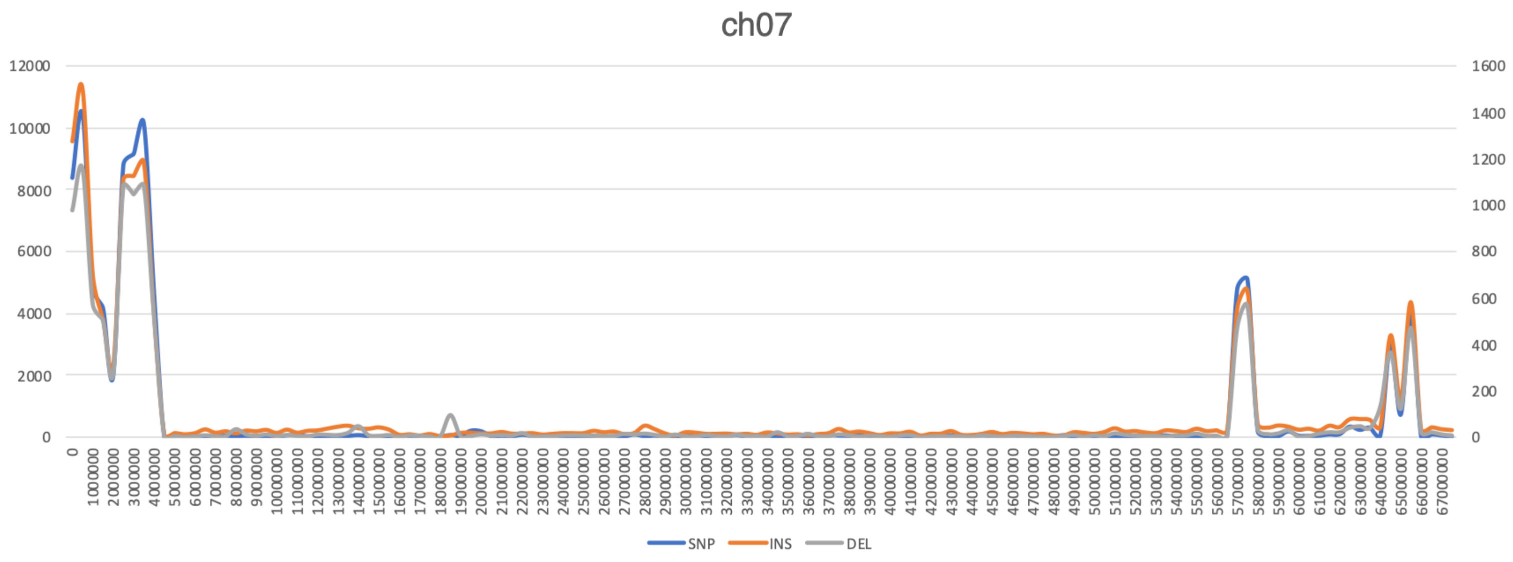

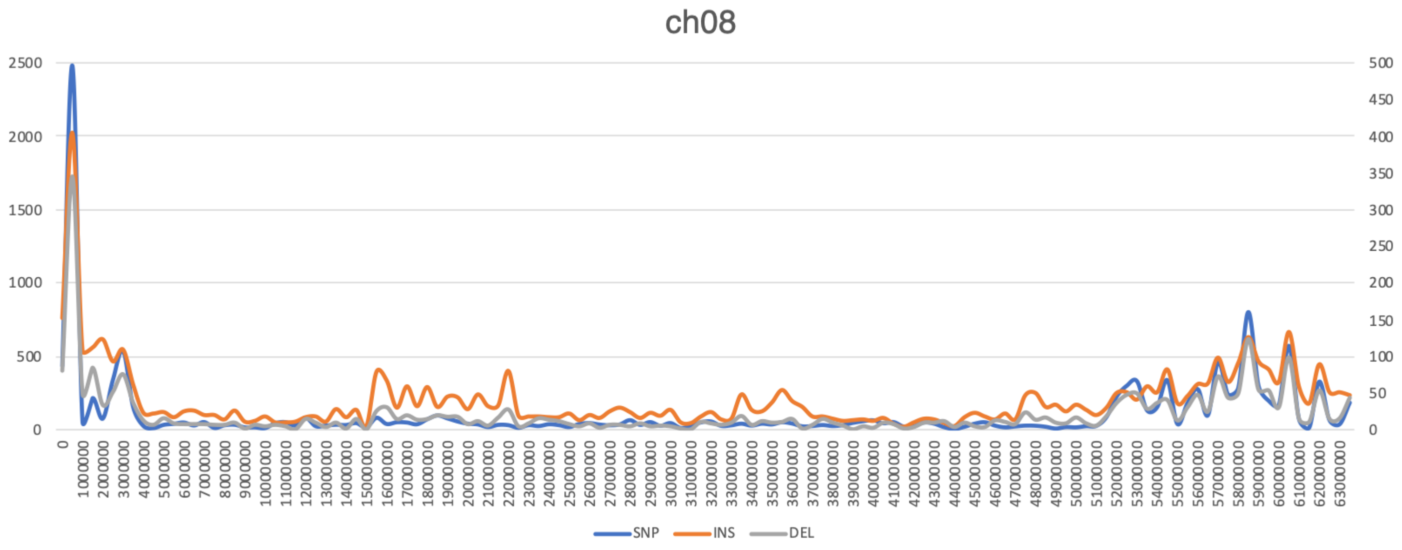

Supplementary Figure S4a

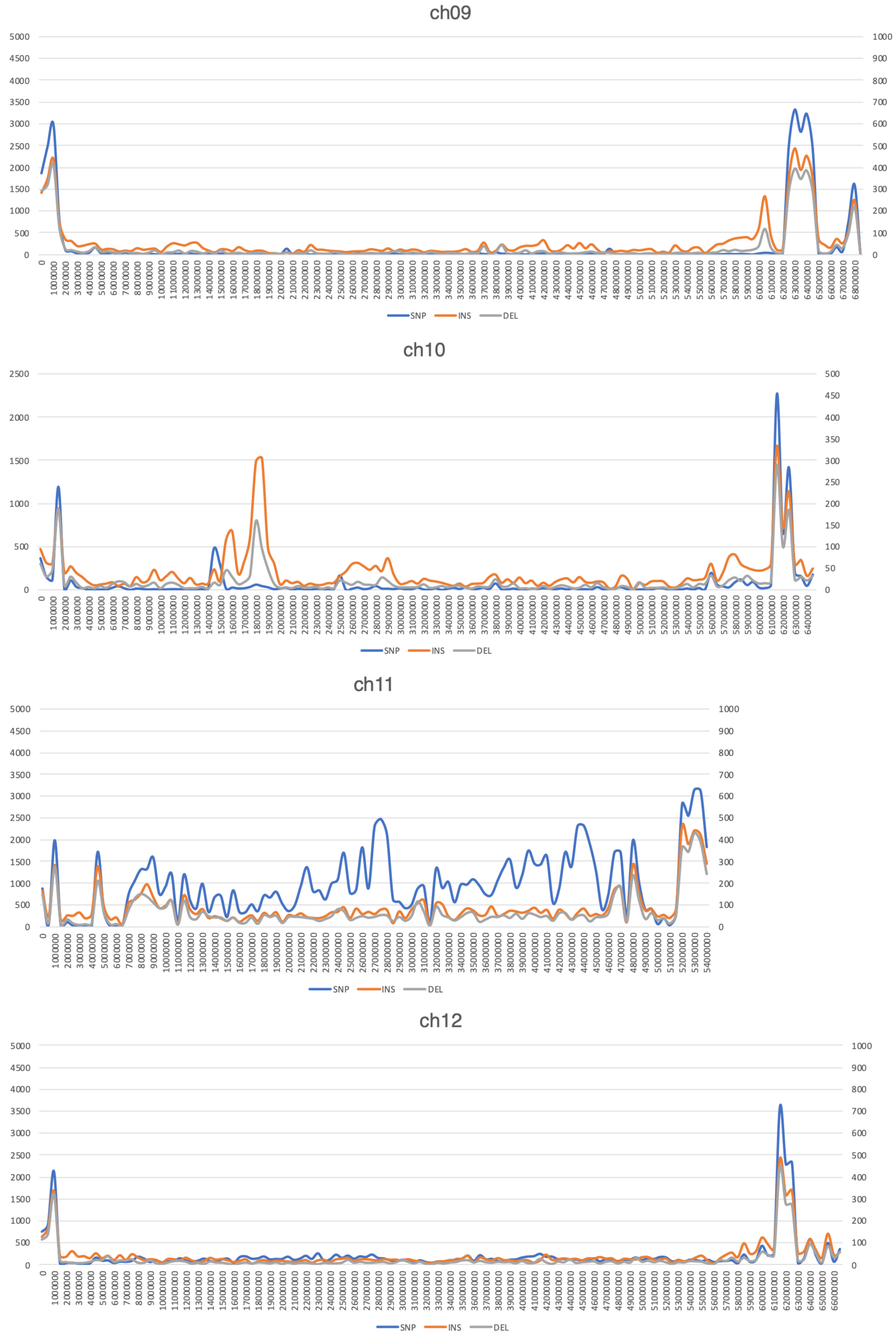

Supplementary Figure S4a

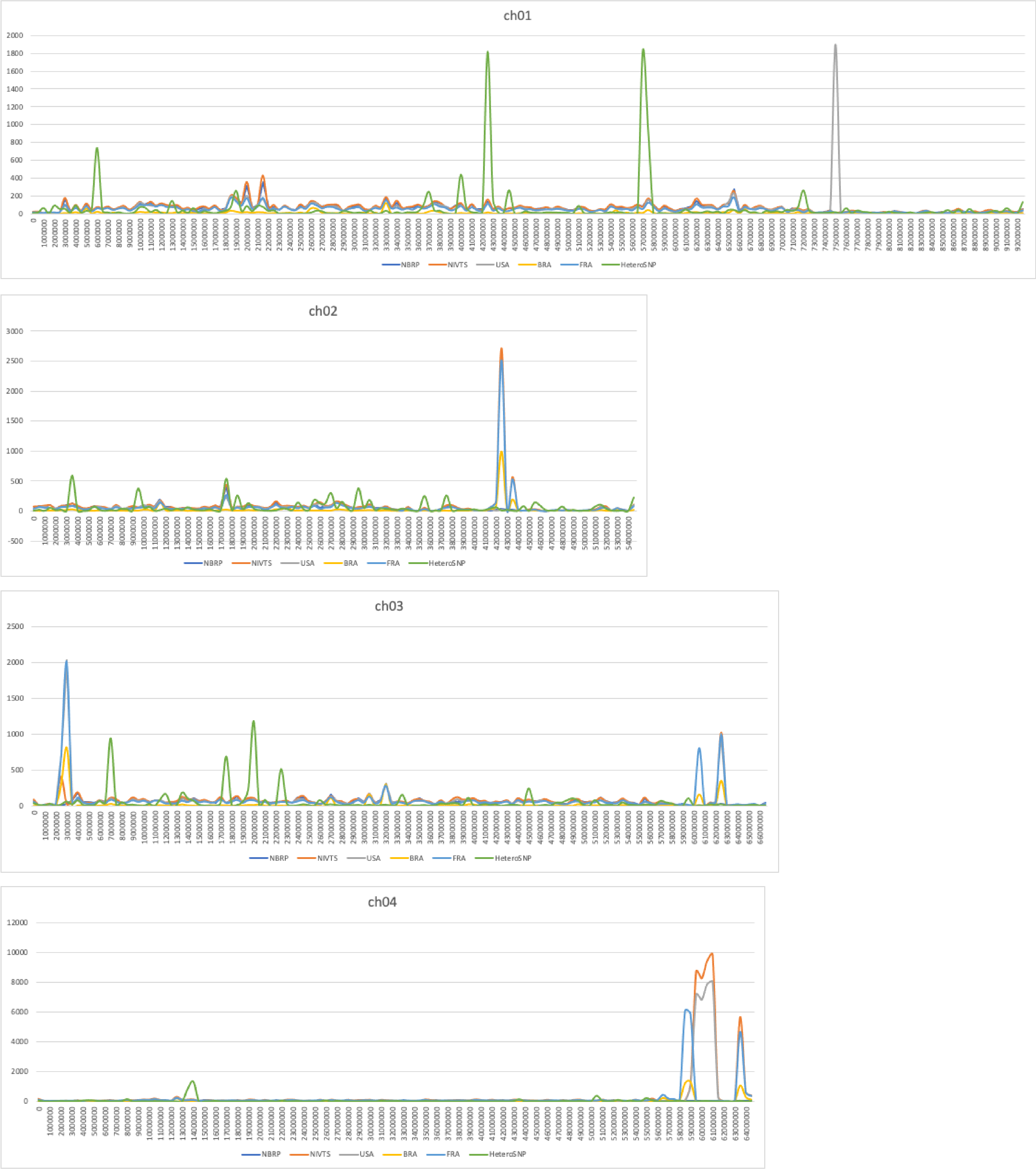

Supplementary Figure S4b. Chromosomal distribution of SNPs between Micro-Tom S9 (KDRI line) and other close lines, and heterozygous SNPs of KDRI line.

The horizontal line represents the physical distance along respective chromosome, split into 500 kb windows. The right vertical line indicates the number of SNPs. Respective line colored blue, orange, grey, yellow, and blue indicates SNPs against KDRI with NBRP, NIVTS, USA, BRA, and FRA. The green line indicates heterozygous SNPs of KDRI line.

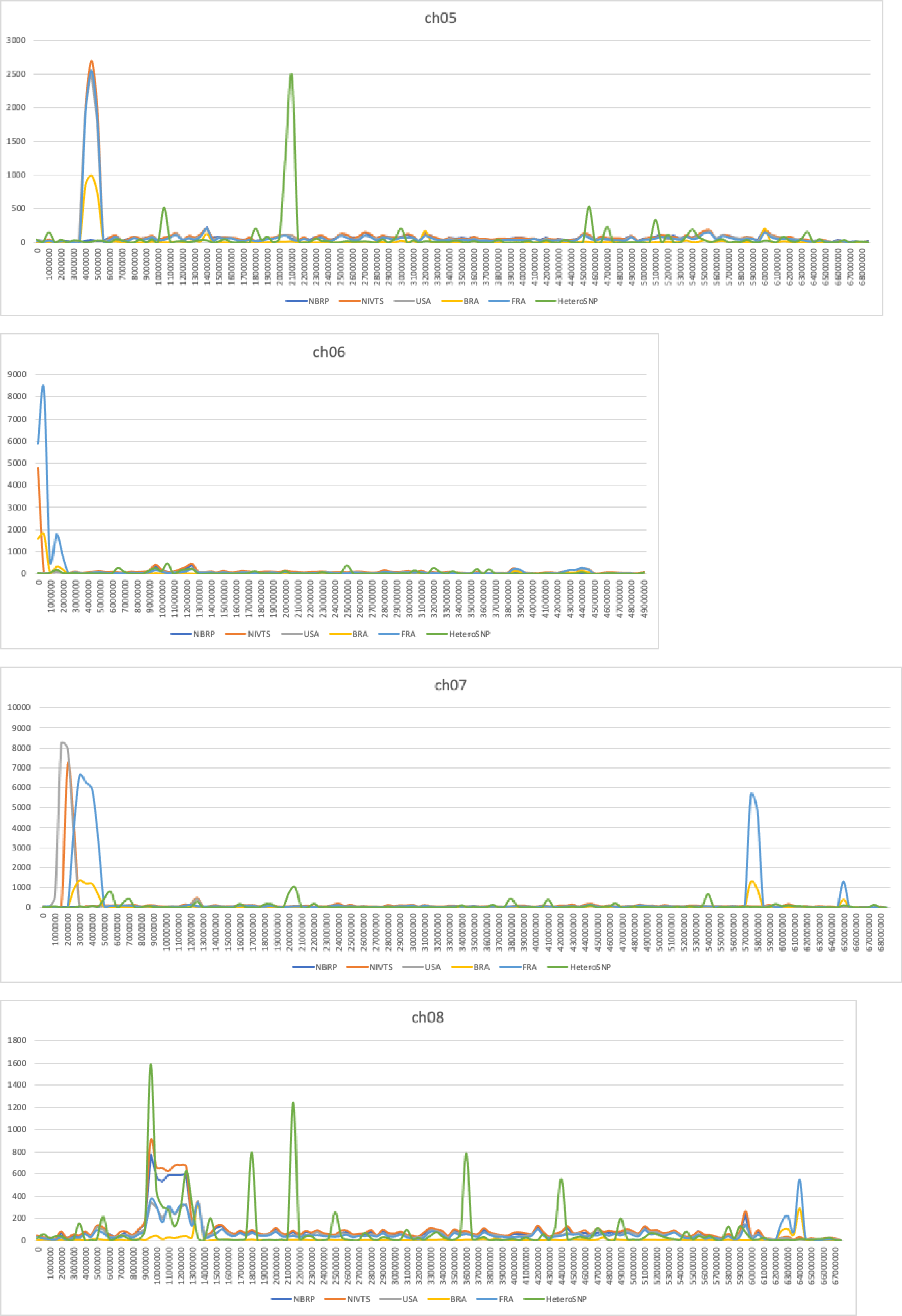

Supplementary Figure S4b

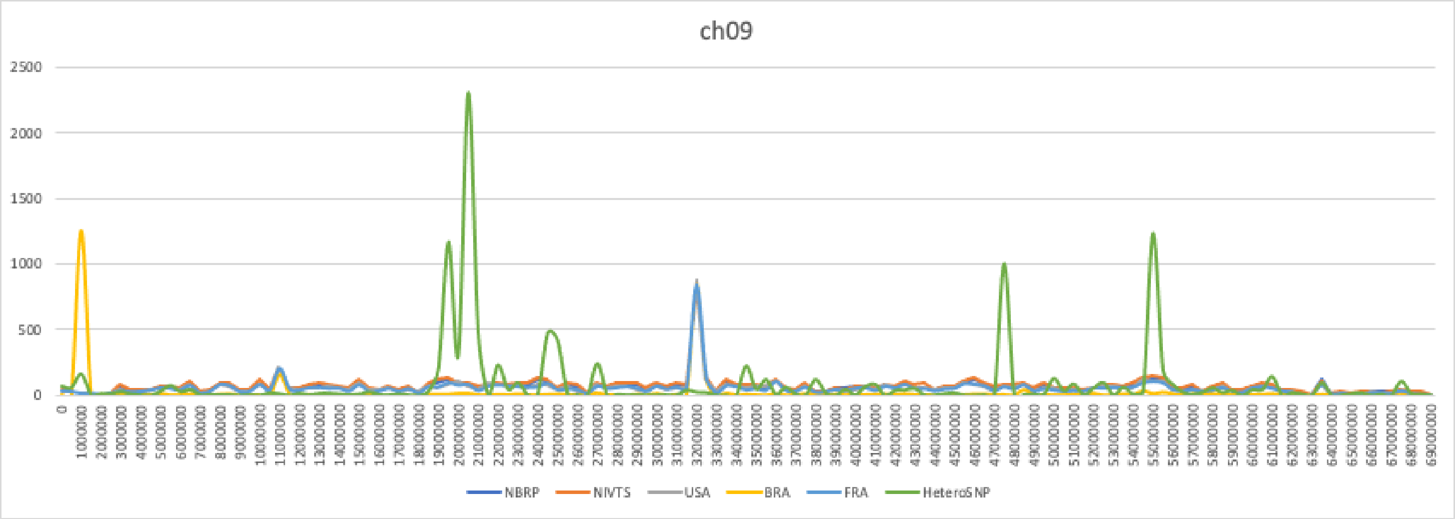

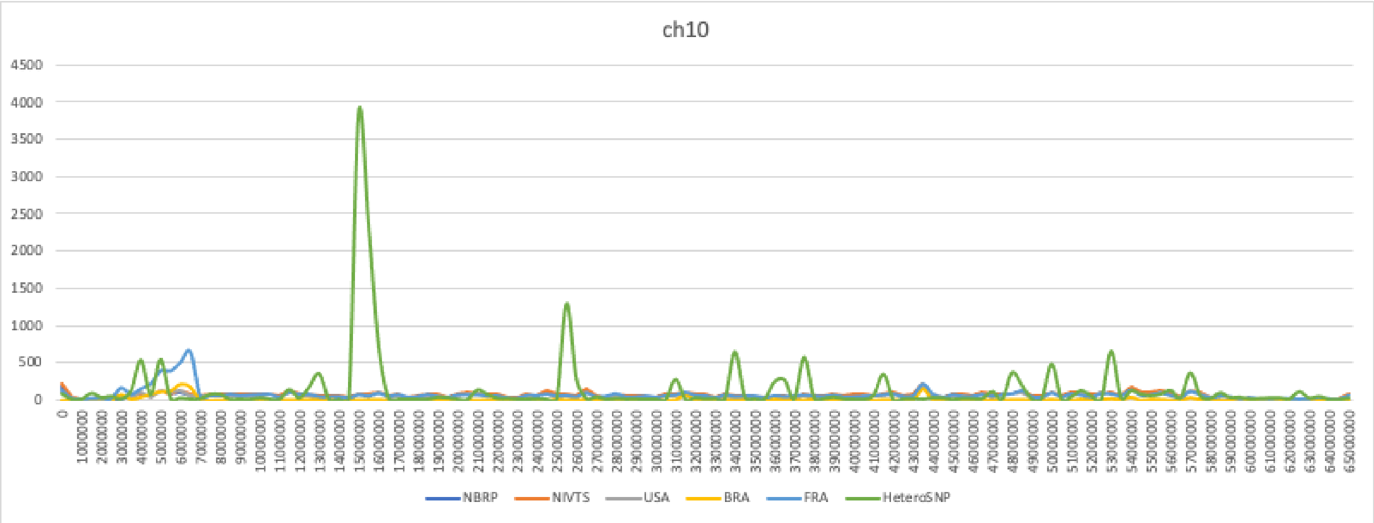

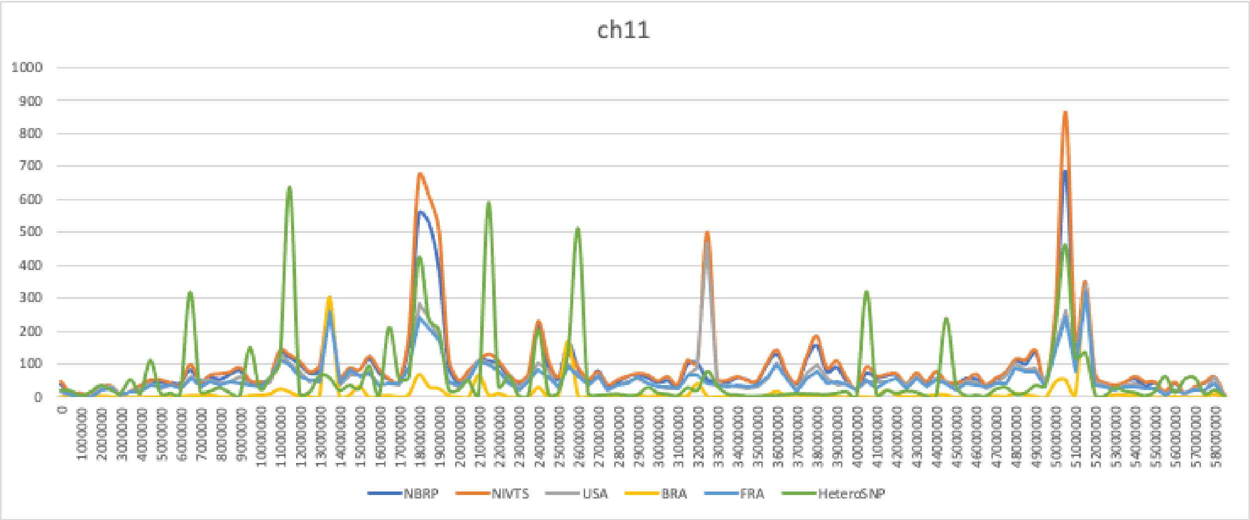

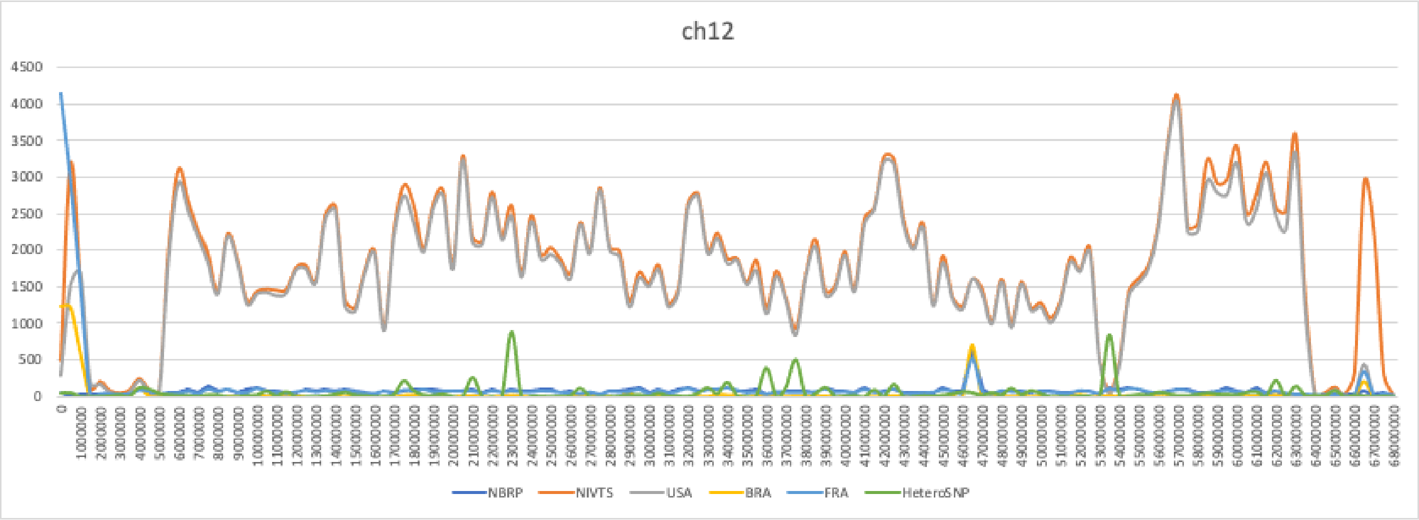

Supplementary Figure S4b

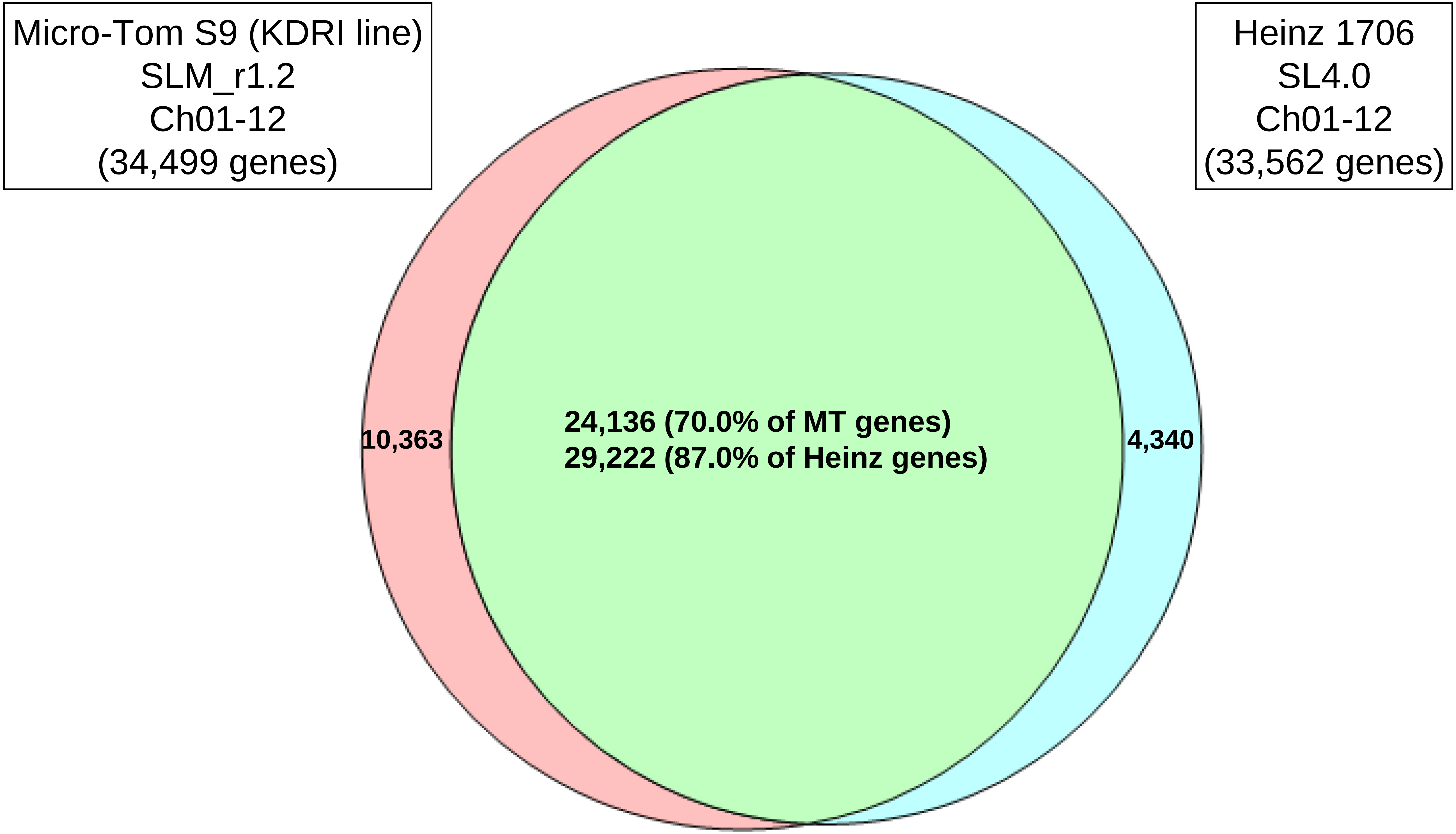

Supplementary Figure S5. The share of predicted genes that matched between Micro-Tom KDRI line and Heinz 1706 by genomic positions.
Predicted genes of Micro-Tom and ITAG 4.0 gene set of Heinz, those were compared by the located/mapped genomic positions on ch01 to ch12 of SLM_r1.2 Micro-Tom genome.

A)

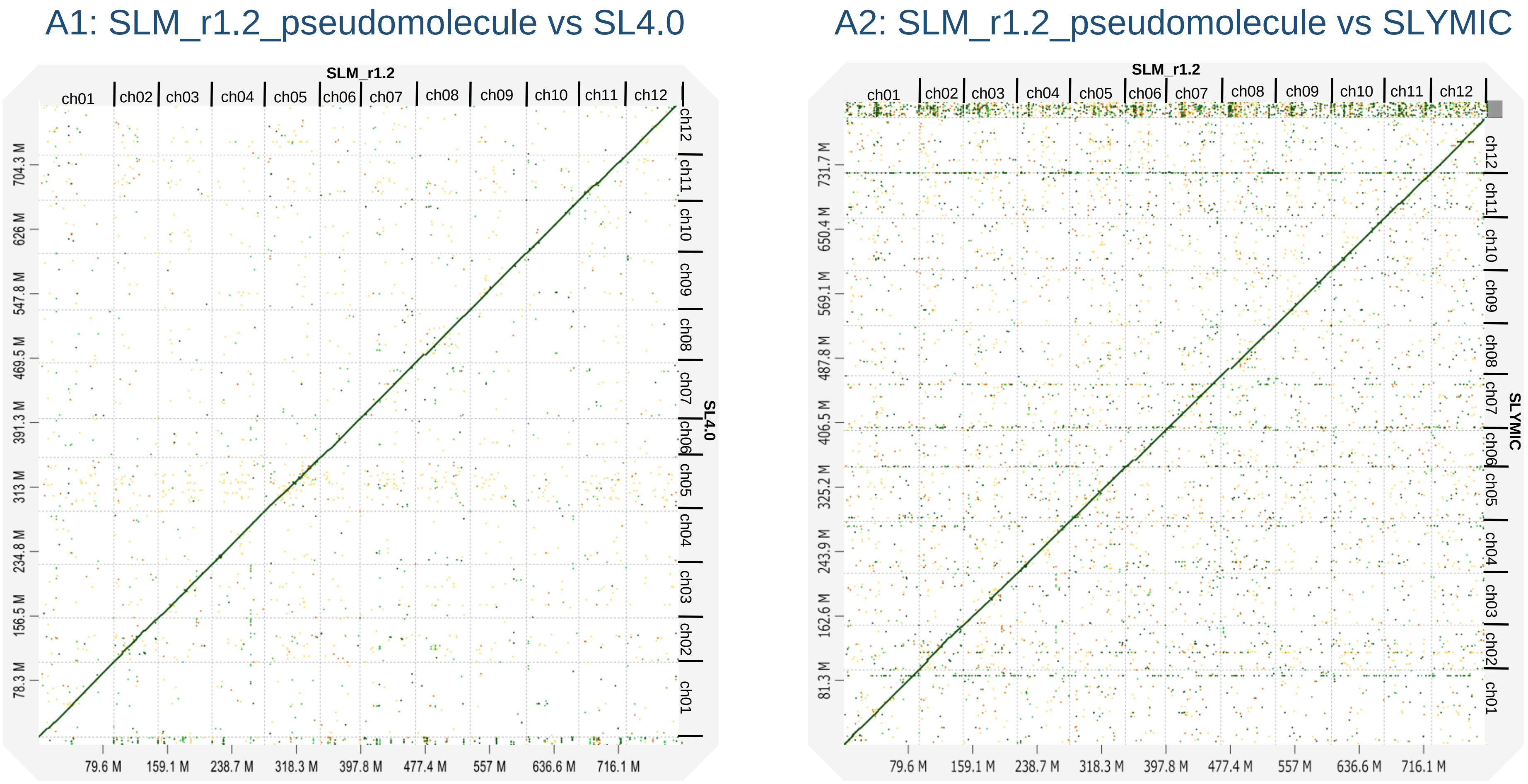

Supplementary Figure S6. Genomic alignment between SLM_r1.2 and SL4.0 (A1: horizontal axis represents SLM_r1.2, vertical axis represents SL4.0) and between SLM_r1.2 and SLYMIC (A2: horizontal axis represents SLM_r1.2, vertical axis represents SLYMIC). Figure A represents alignments of whole genome. B to M represent alignments of respective chromosome.

B)

B1: SLM_r1.2_pseudomolecule vs SL4.0 (ch01)

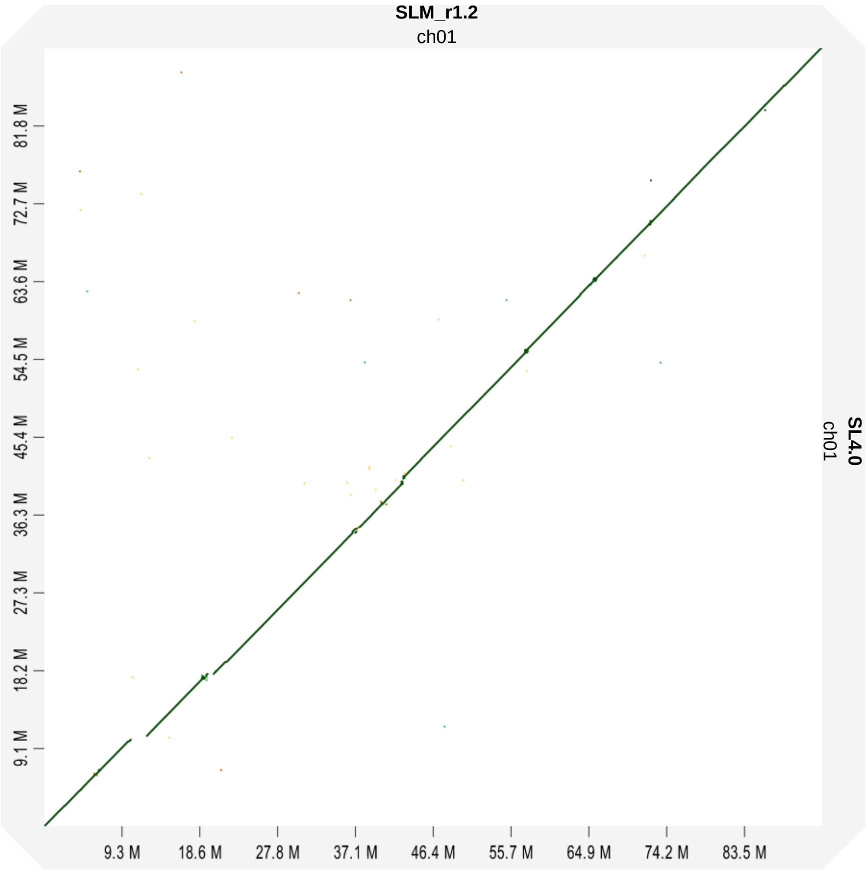

B2: SLM_r1.2_pseudomolecule vs SLYMIC (ch01)

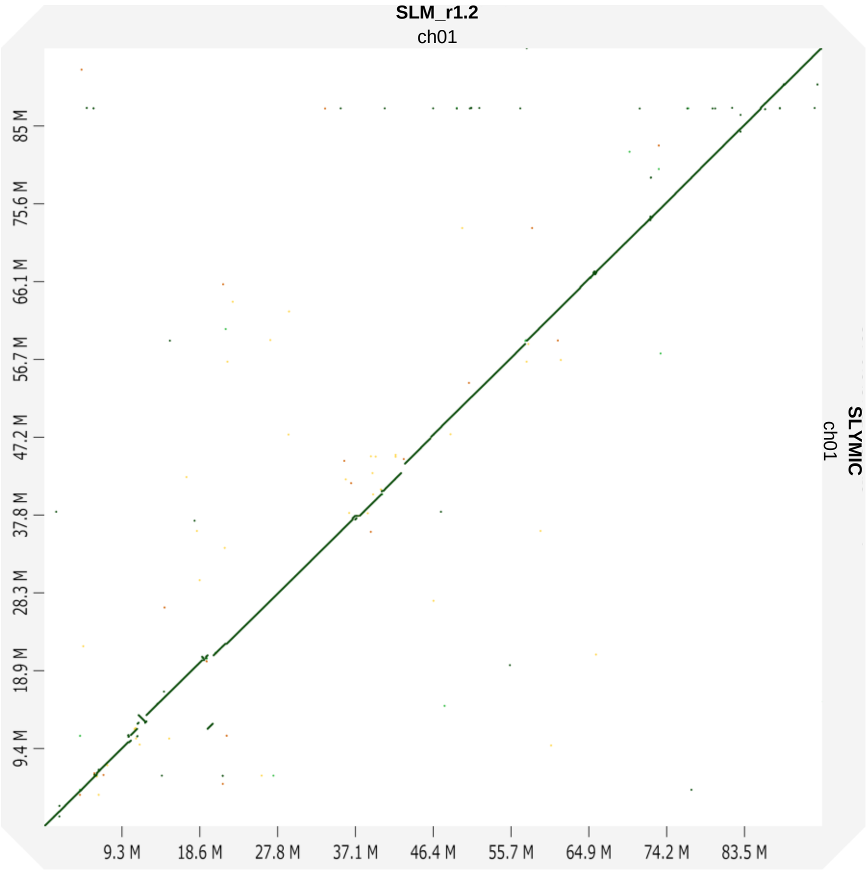

C)

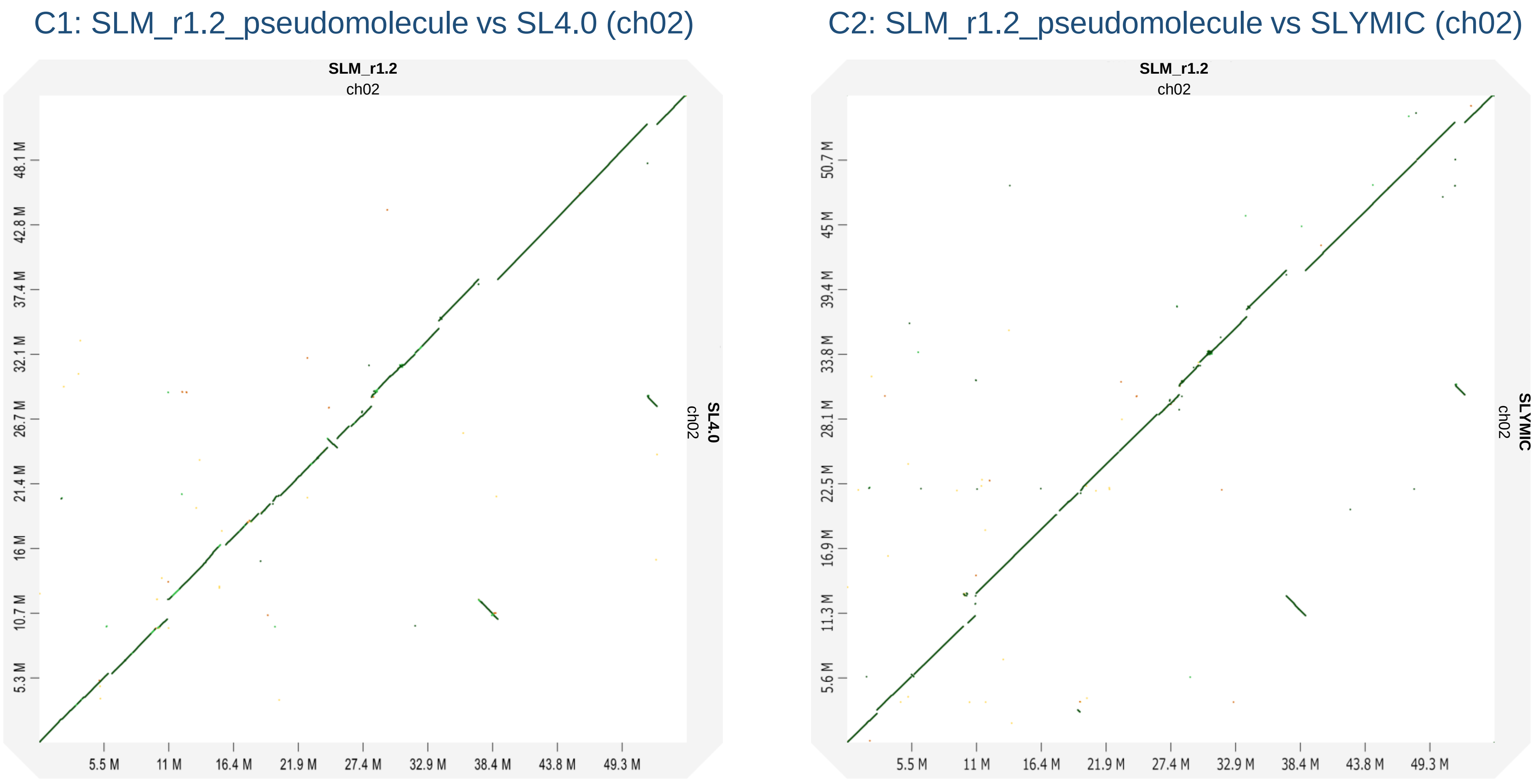
C1: SLM_r1.2_pseudomolecule vs SL4.0 (ch02)
SLM_r1.2
ch02
SL4.0
ch02
48.1 M
42.8 M
37.4 M
32.1 M
26.7 M
21.4 M
16 M
10.7 M
5.3 M
5.5 M
11 M
16.4 M
21.9 M
27.4 M
32.9 M
38.4 M
43.8 M
49.3 M
C2: SLM_r1.2_pseudomolecule vs SLYMIC (ch02)
SLM_r1.2
ch02
SLYMIC
ch02
50.7 M
45 M
39.4 M
33.8 M
28.1 M
22.5 M
16.9 M
11.3 M
5.6 M
5.5 M
11 M
16.4 M
21.9 M
27.4 M
32.9 M
38.4 M
43.8 M
49.3 M

D)

D1: SLM_r1.2_pseudomolecule vs SL4.0 (ch03)

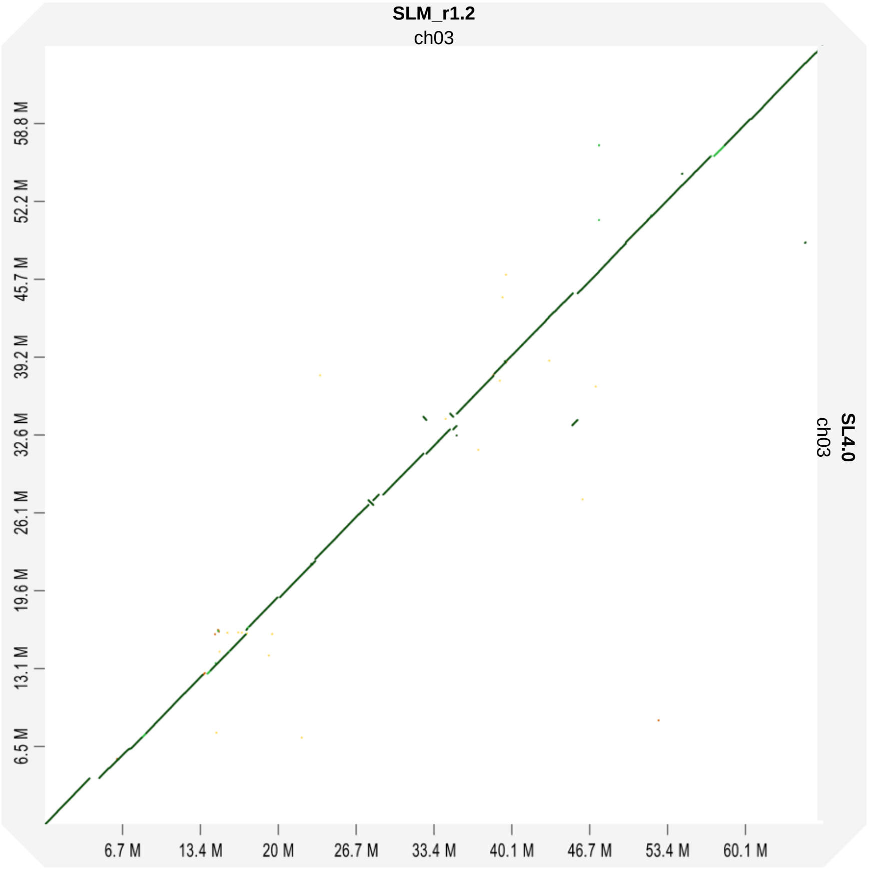

D2: SLM_r1.2_pseudomolecule vs SLYMIC (ch03)

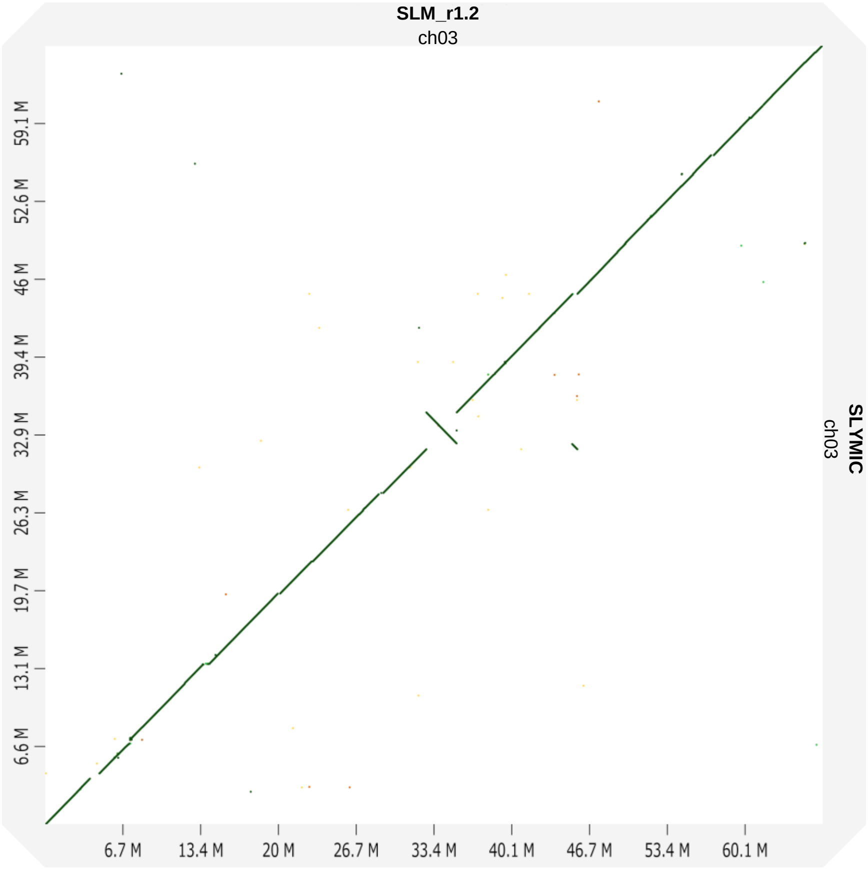

E)

E1: SLM_r1.2_pseudomolecule vs SL4.0 (ch04)

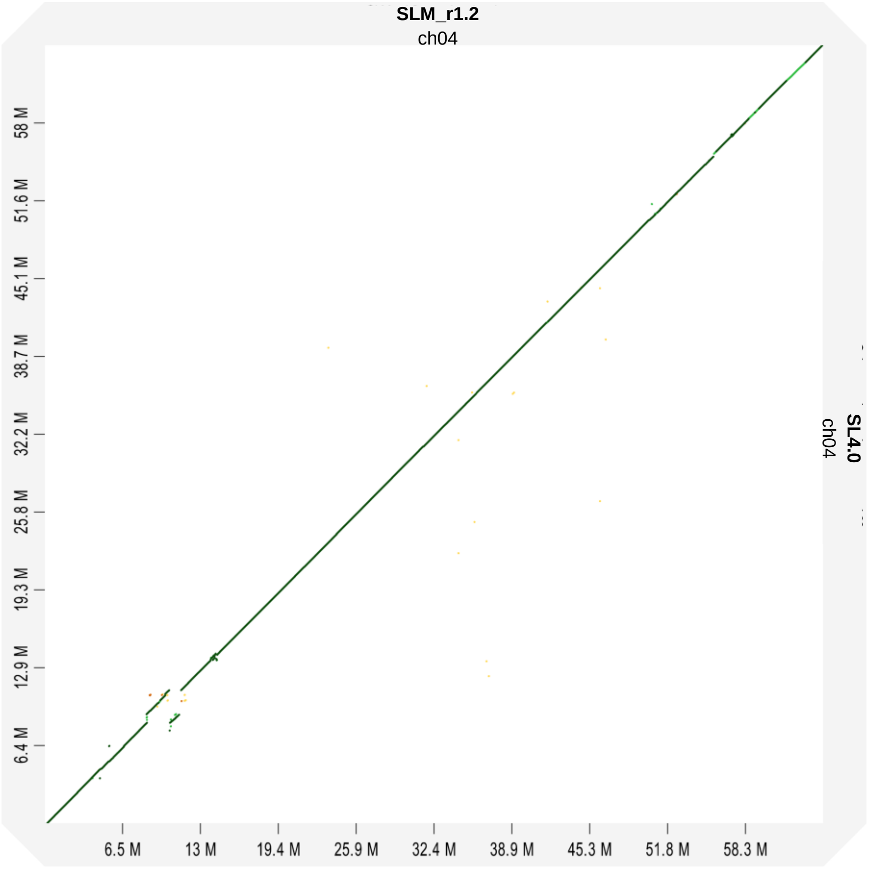

E2: SLM_r1.2_pseudomolecule vs SLYMIC (ch04)

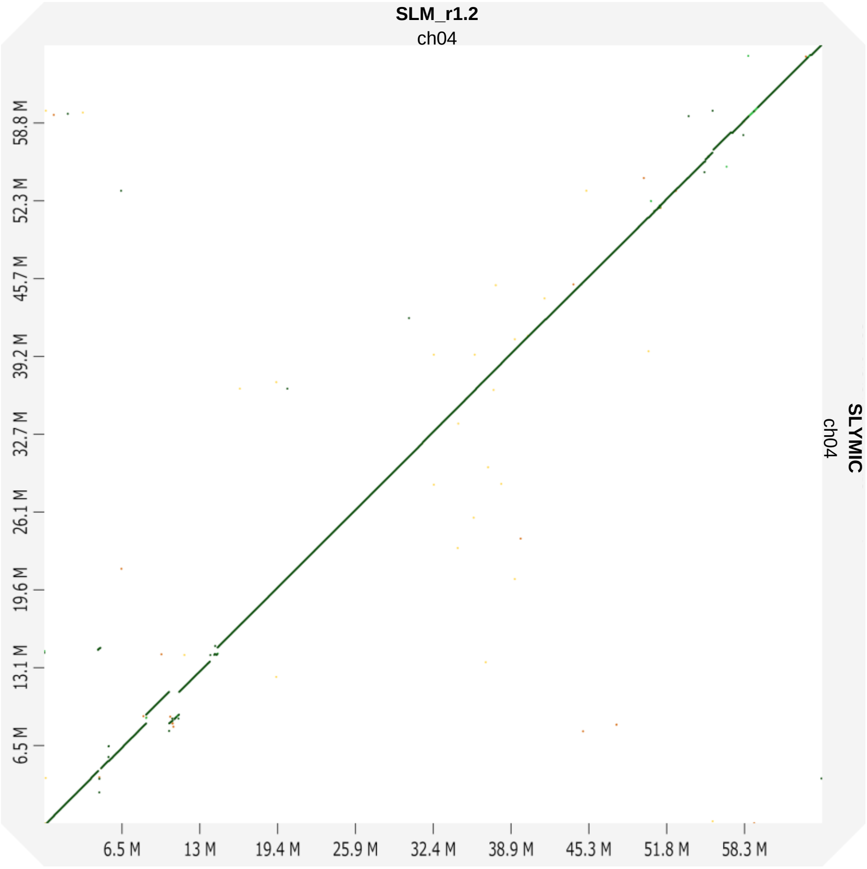

F)

F1: SLM_r1.2_pseudomolecule vs SL4.0 (ch05)

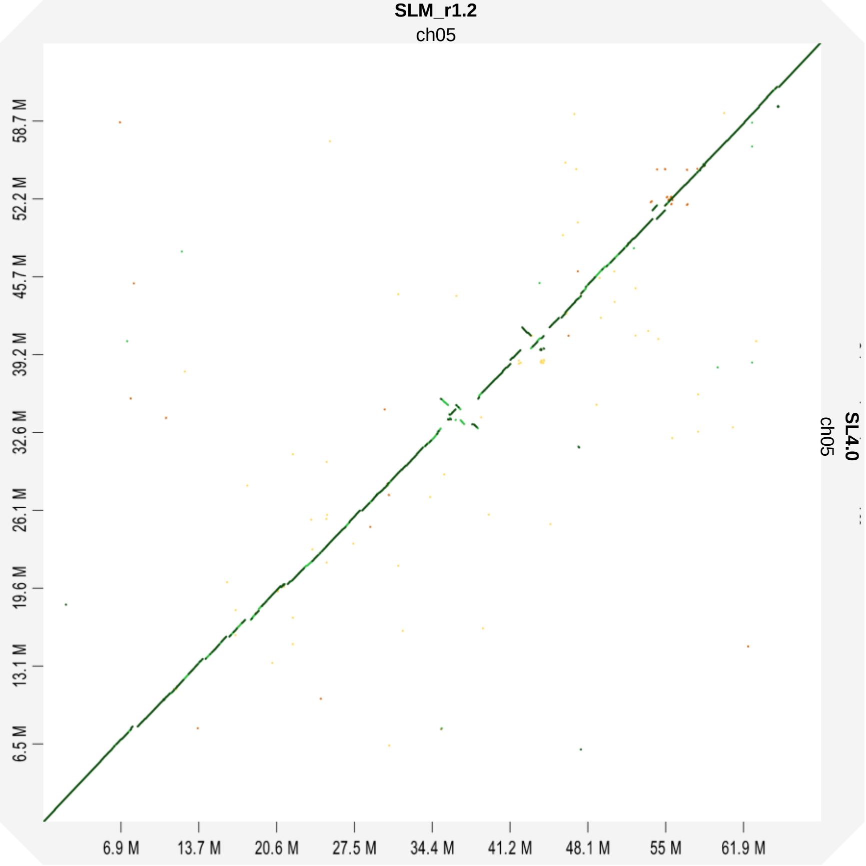

F2: SLM_r1.2_pseudomolecule vs SLYMIC (ch05)

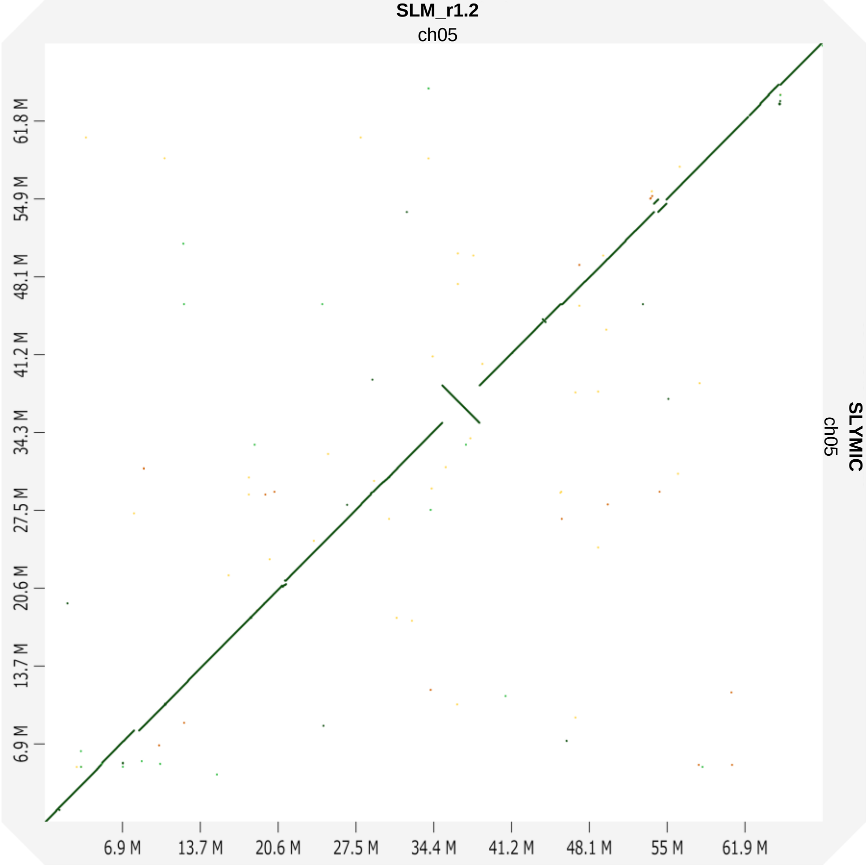

G)

G1: SLM_r1.2_pseudomolecule vs SL4.0 (ch06)

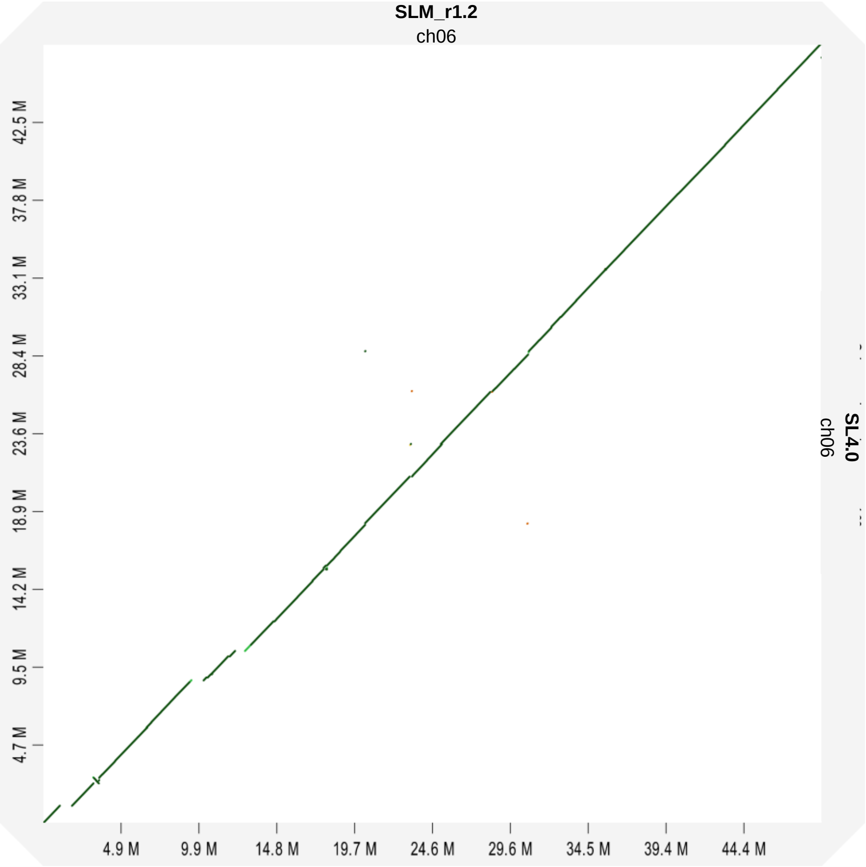

G2: SLM_r1.2_pseudomolecule vs SLYMIC (ch06)

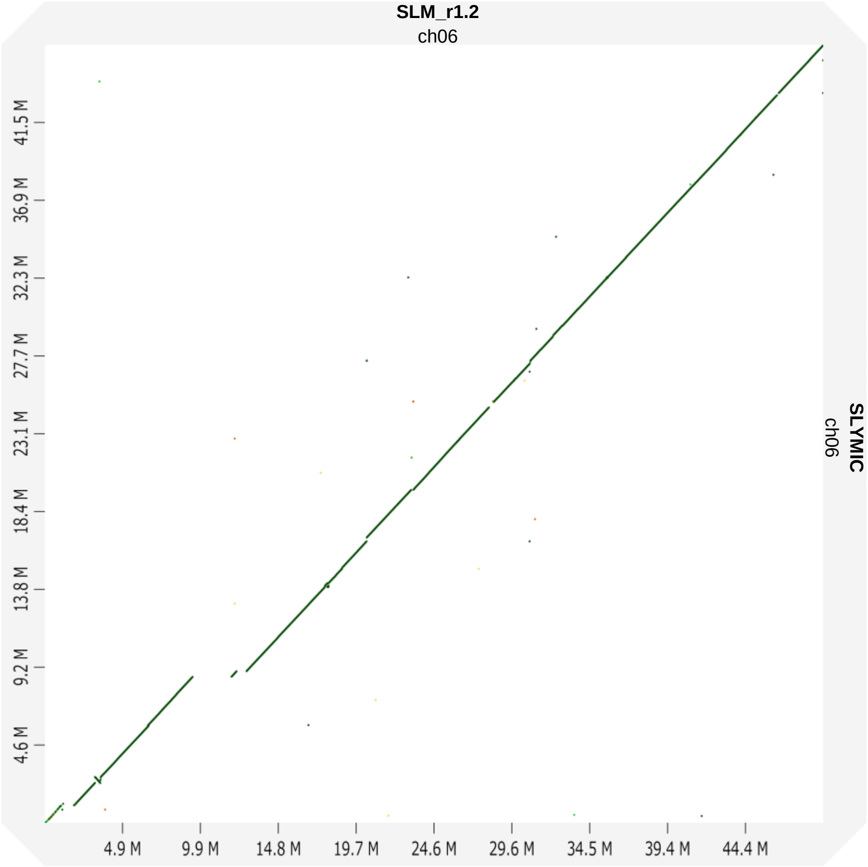

H)

H1: SLM_r1.2_pseudomolecule vs SL4.0 (ch07)

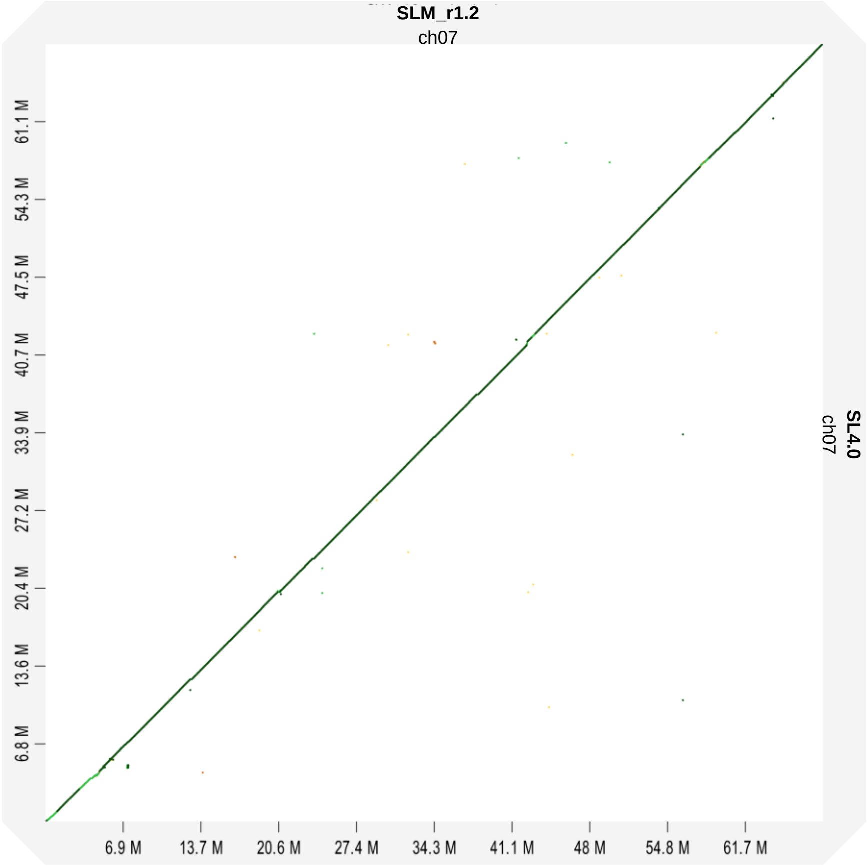

H2: SLM_r1.2_pseudomolecule vs SLYMIC (ch07)

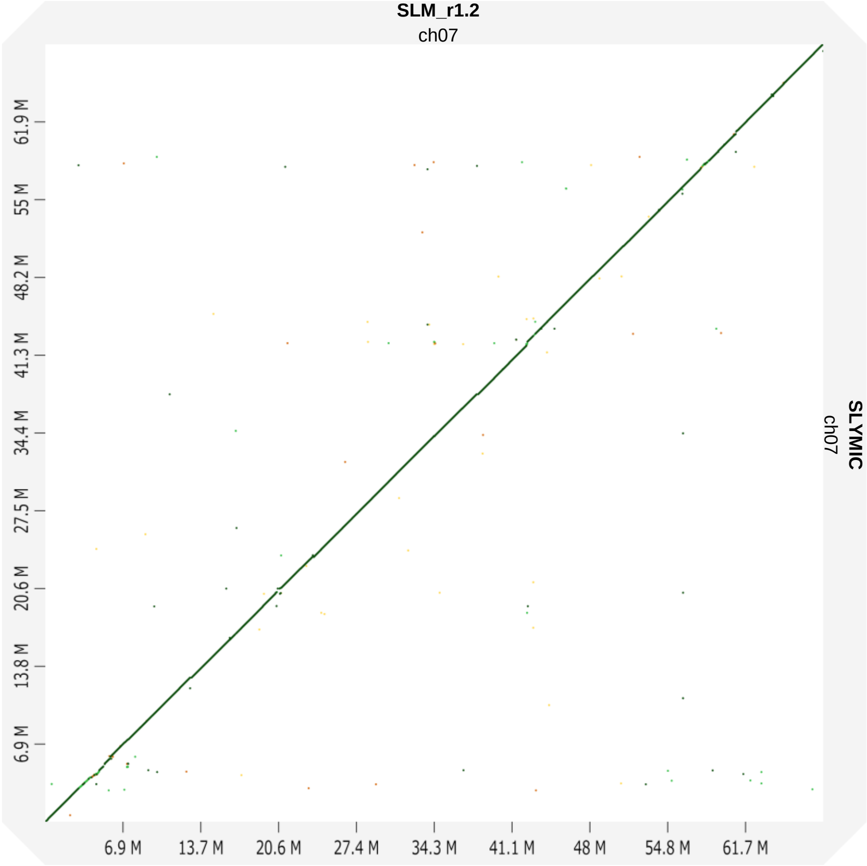

l)

I1: SLM_r1.2_pseudomolecule vs SL4.0 (ch08)

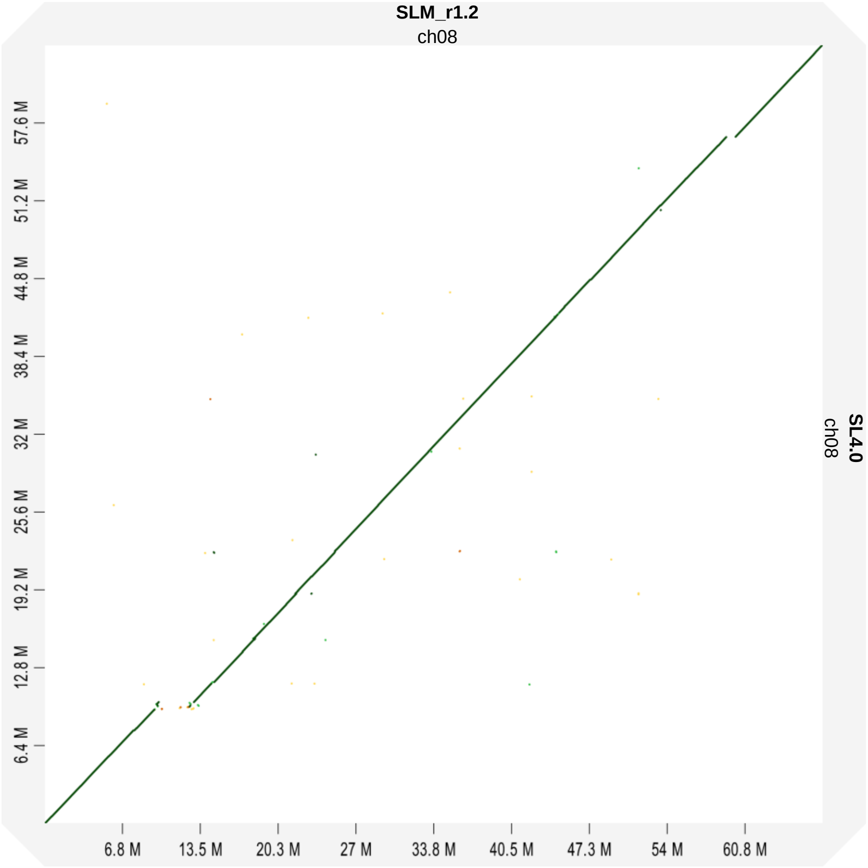

I2: SLM_r1.2_pseudomolecule vs SLYMIC (ch08)

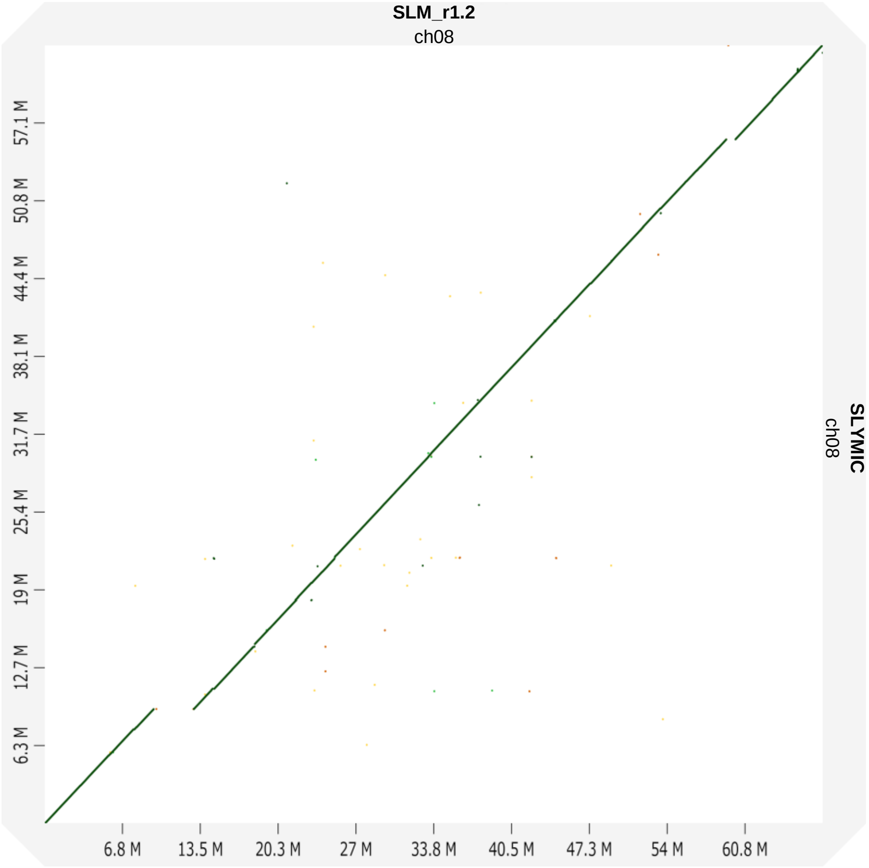

J)

J1: SLM_r1.2_pseudomolecule vs SL4.0 (ch09)

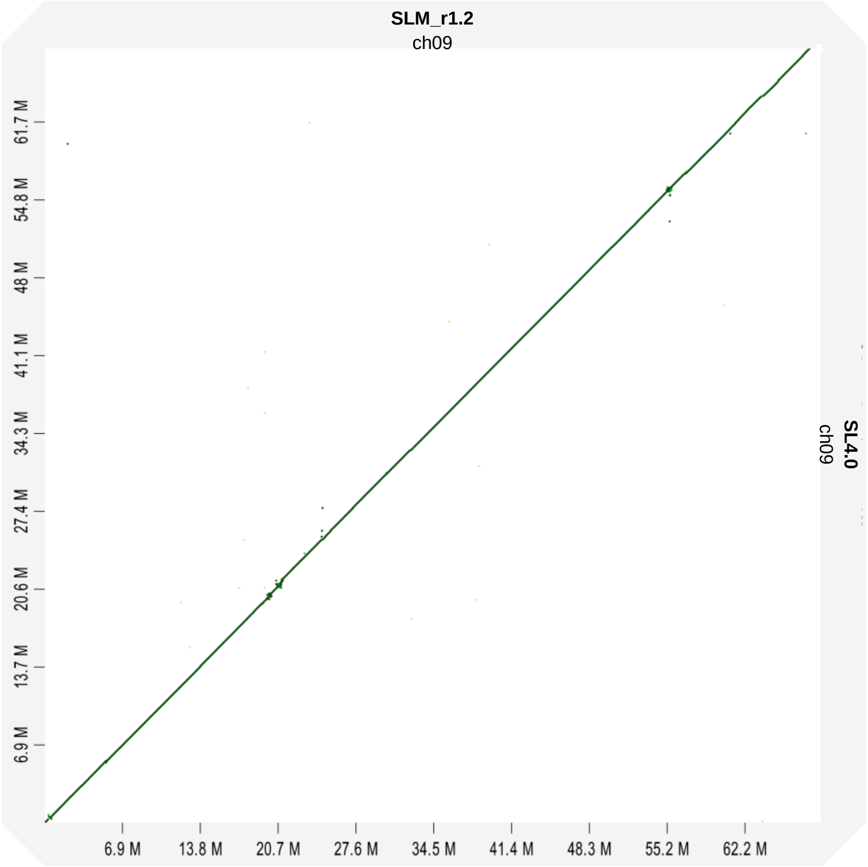

J2: SLM_r1.2_pseudomolecule vs SLYMIC (ch09)

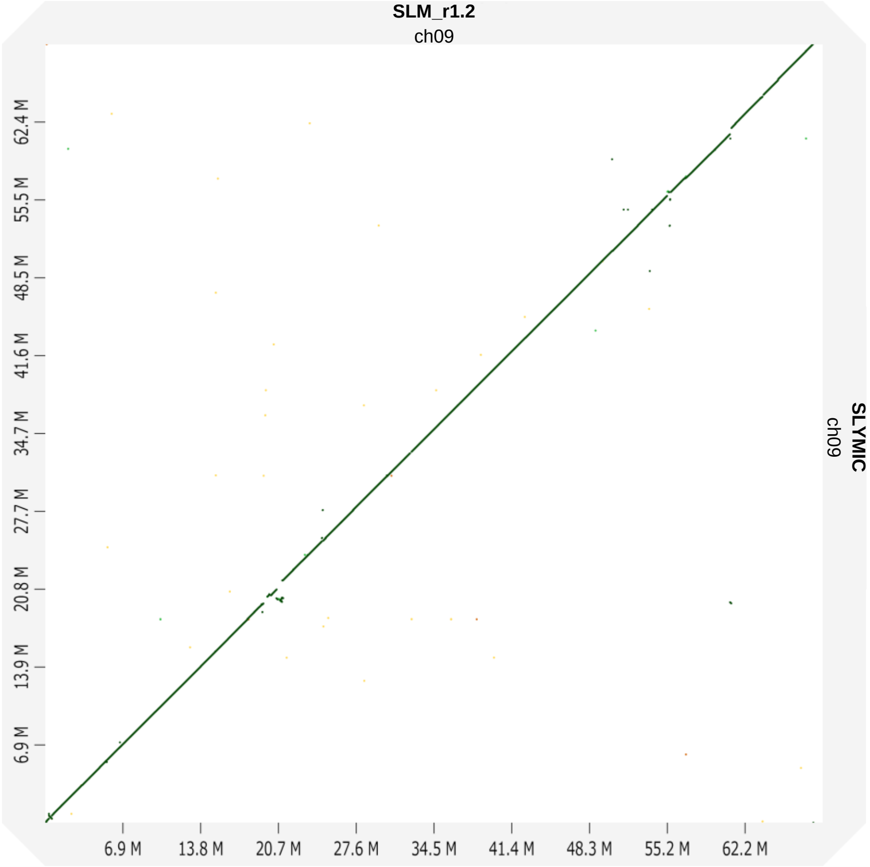

K)

K1: SLM_r1.2_pseudomolecule vs SL4.0 (ch10)

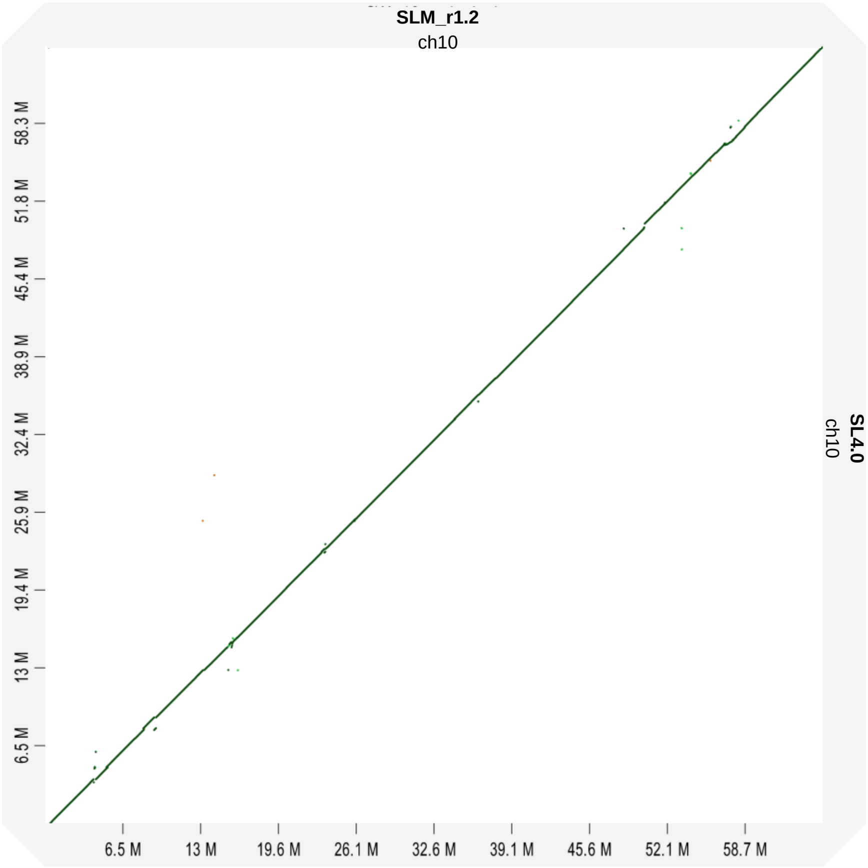

K2: SLM_r1.2_pseudomolecule vs SLYMIC (ch10)

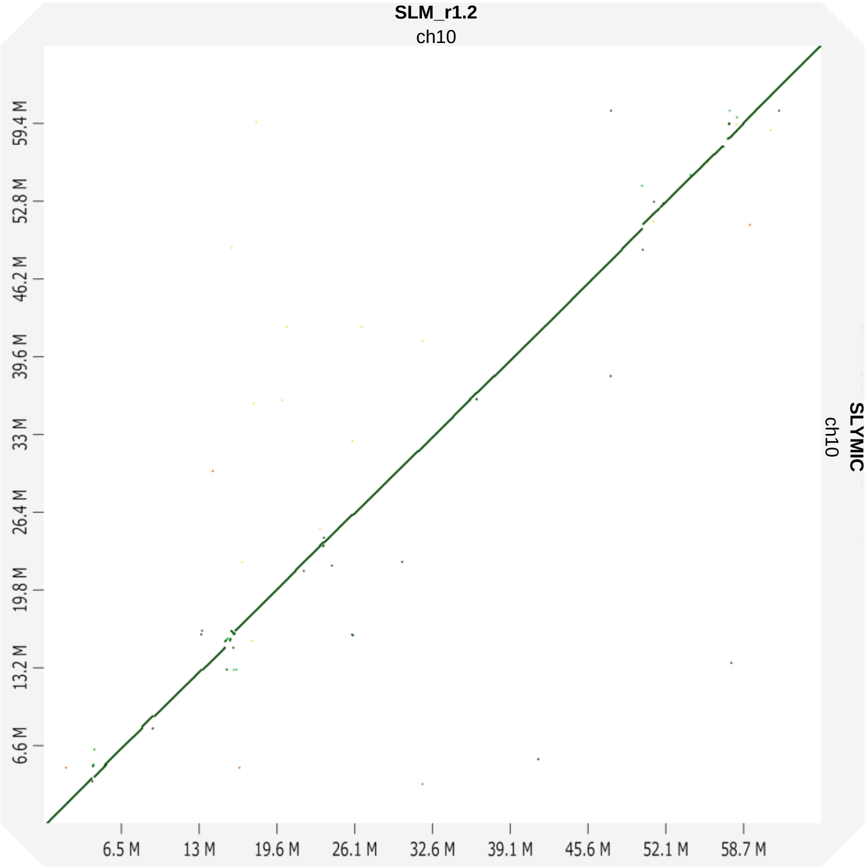

L)

L1: SLM_r1.2_pseudomolecule vs SL4.0 (ch11)

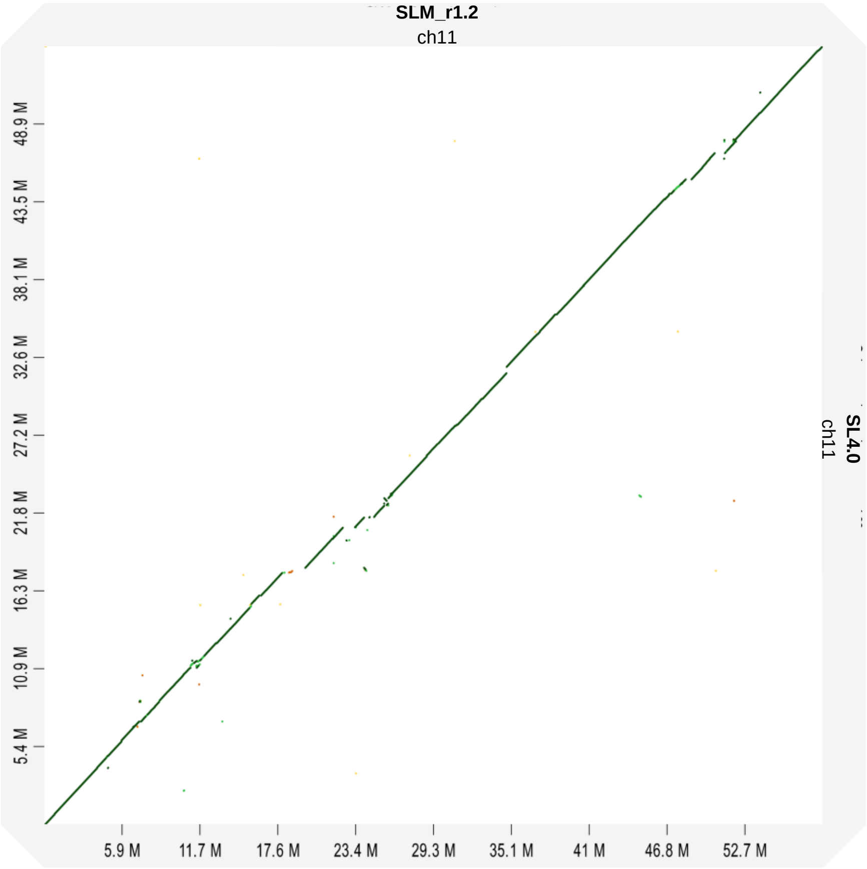

L2: SLM_r1.2_pseudomolecule vs SLYMIC (ch11)

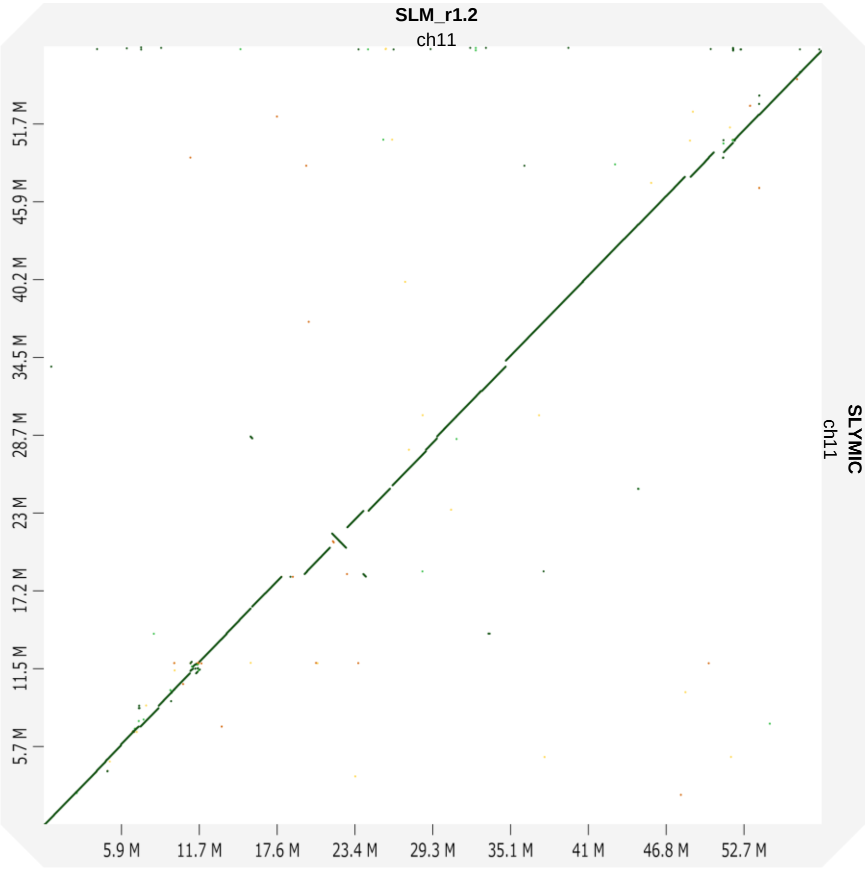

M)

M1: SLM_r1.2_pseudomolecule vs SL4.0 (ch12)

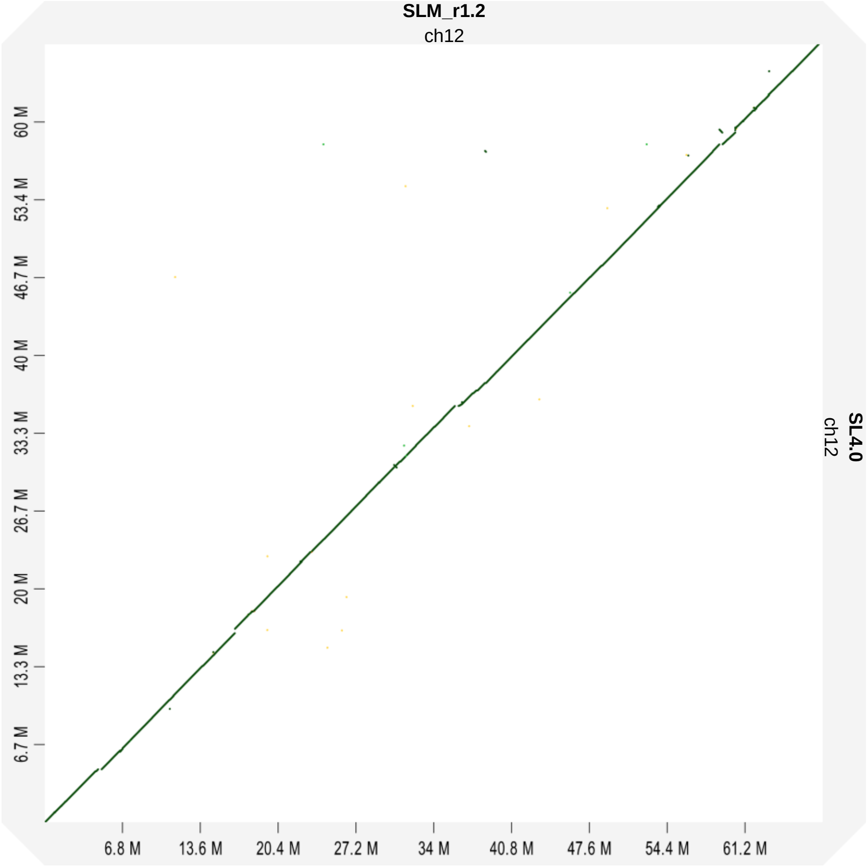

M2: SLM_r1.2_pseudomolecule vs SLYMIC (ch12)

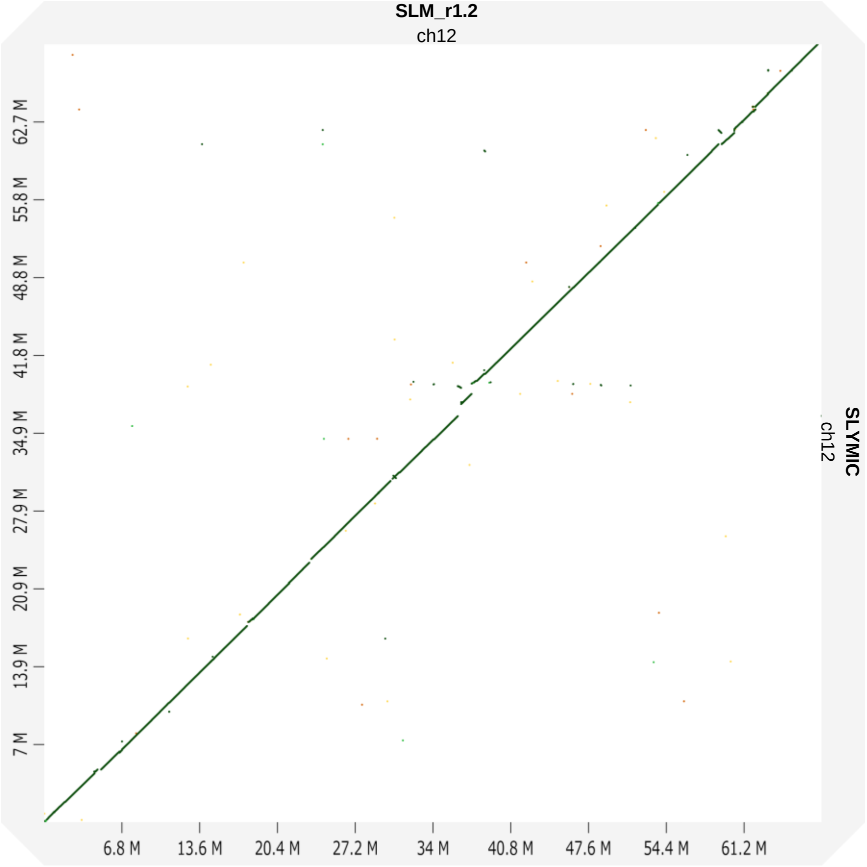

Supplement: dsae016_suppl_Supplementary_Figures [file dsae016_suppl_supplementary_figures.pdf]
