# Supplementary material for: Genomic variation across distribution of Micro-Tom, a model cultivar of tomato (Solanum lycopersicum)
Source: DNA Res. 2024 Jun 7;31(5):dsae016. doi: 10.1093/dnares/dsae016 (PMC11481021; doi:10.1093/dnares/dsae016)
Supplement: dsae016_suppl_Supplementary_Data_S1 [file dsae016_suppl_supplementary_data_s1.pdf]

Supplementary Data 1. Continuous phenotypic alteration of six Micro-Tom lines and CAPs markers to detect genetic polymorphism

Materials and methods

1. Plant materials

In a greenhouse at University of Tsukuba (Tsukuba, Japan), seeds were sown in 5 cm diameter pots filled with JIFFY mix (Sakata Seed Corp., Japan). After buds on the plants appeared, all the plants were transplanted to 20 cm diameter pots and watered every morning and were provided with Hyponex nutrient solution (Hyponex, Japan) weekly at a concentration of 0.2% before anthesis and 0.5% after anthesis. NBRP, NIVITS, USA, BRA, and FRA lines in a greenhouse were cultivated for 105 days, from seed sowing to fruit harvesting (from Mar. 13 to Jun. 25, 2018) and KDRI line in a greenhouse was cultivated for 90 days, from seed sowing to fruit harvesting (from Mar. 27 to Jun. 25, 2018). In the cultivation room, seeds were sown on wet filter papers in a petri dish moistened with deionized water at 25 ℃ (16 h/8 h light/dark, 60.0 μmol m-2 s-1). After 1 week of germination, seedlings that germinated normally were transplanted to hydroculture medium Rockwool cubes (Grodan), and Otsuka 1 Gou and 2 Gou nutrient solutions (OAT Agrio Co., Ltd., Japan) were supplied. NBRP, NIVITS, USA, BRA, and FRA lines in a greenhouse were cultivated for 115 days, from seed sowing to fruit harvesting (from Mar. 13 to Jul. 5, 2018) and KDRI line in a greenhouse was cultivated for 100 days, from seed sowing to fruit harvesting (from Mar. 27 to Jun. 25, 2018).

2. Development of CAPs markers on each chromosome to detect genetic polymorphism in six Micro-Tom lines.

For evaluation of CAPs markers, FRA line, which has been published most complete genome sequence, was decided as a pistil donor of F_1 hybrids. Each F_1 hybrid was developed by crossing pollen of each line (KDRI, NIVITS, USA, and BRA) to Micro-Tom INRA. All lines were cultivated at 25°C (16 h/8 h light/dark, 60.0 μmol m^{-2} s^{-1}) and F_1 seeds were harvested. All lines and F_1 plants were cultivated at 25°C (16 h/8 h light/dark, 60.0 μmol m^{-2} s^{-1}). Genomic DNAs were extracted from green leaves of each line by a Maxwell 16 Tissue DNA Purification kits using the manufacturer’s protocol (Promega, Tokyo, Japan).

The CAPs primers were designed by each DNA fragment was amplified using each CAPs primer by PCR, and the PCR program consisted as follows: 40 cycles of 10 seconds at 98 ℃, 5 seconds at 58 ℃, 5 seconds at 68 ℃. Each amplified product was digested with each restriction enzyme at 37 ℃ for overnight (Supplementary Data1-1). After that, the digested PCR products were subjected to 2.5 % (w/v) agarose gel electrophoresis (Figure SD1-4).

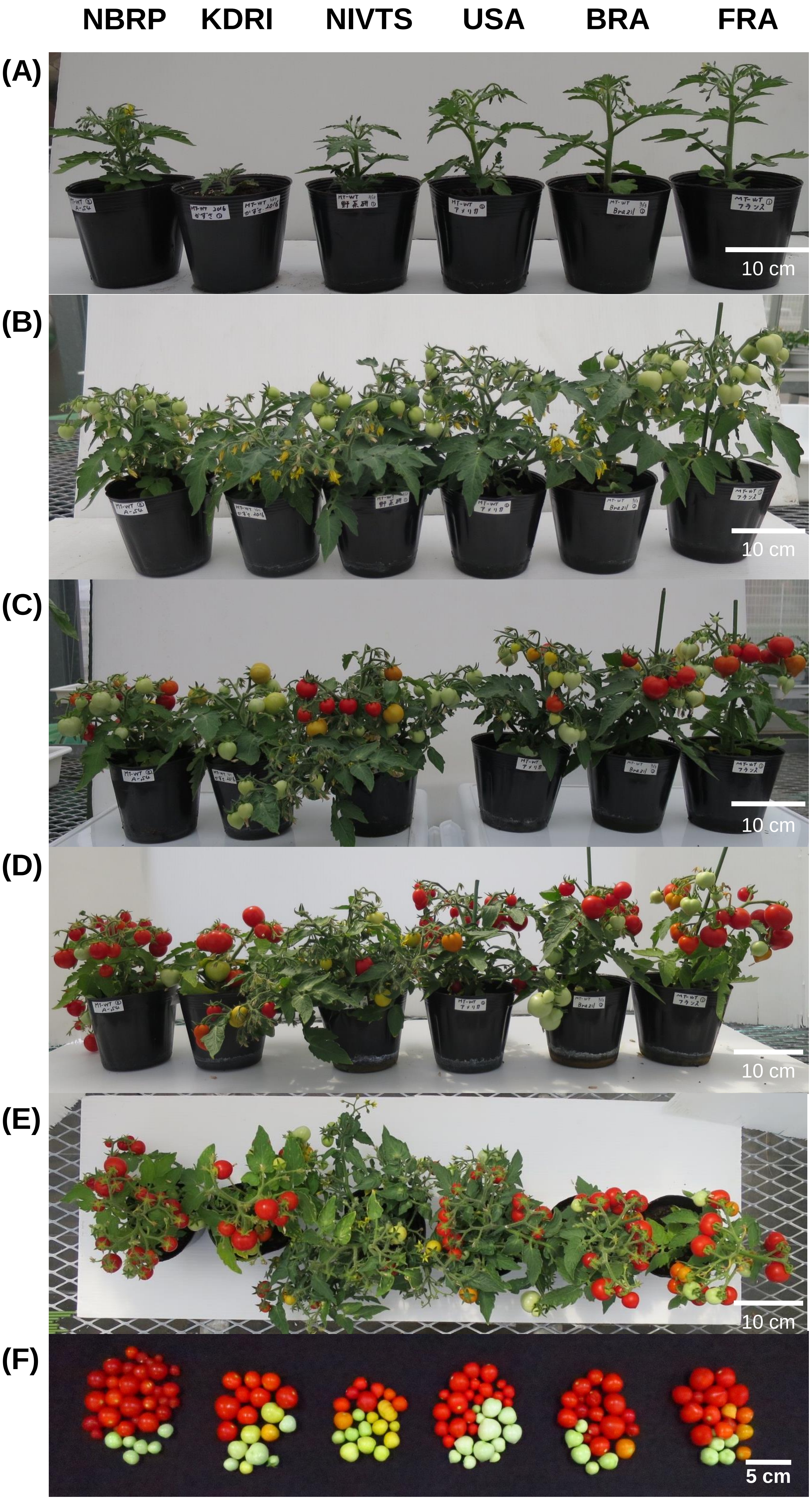

Figure SD1-1. Continuous phenotypic alteration of six Micro-Tom lines (NBRP, KDRI, NIVTS, USA, BRA, and FRA) in a greenhouse.

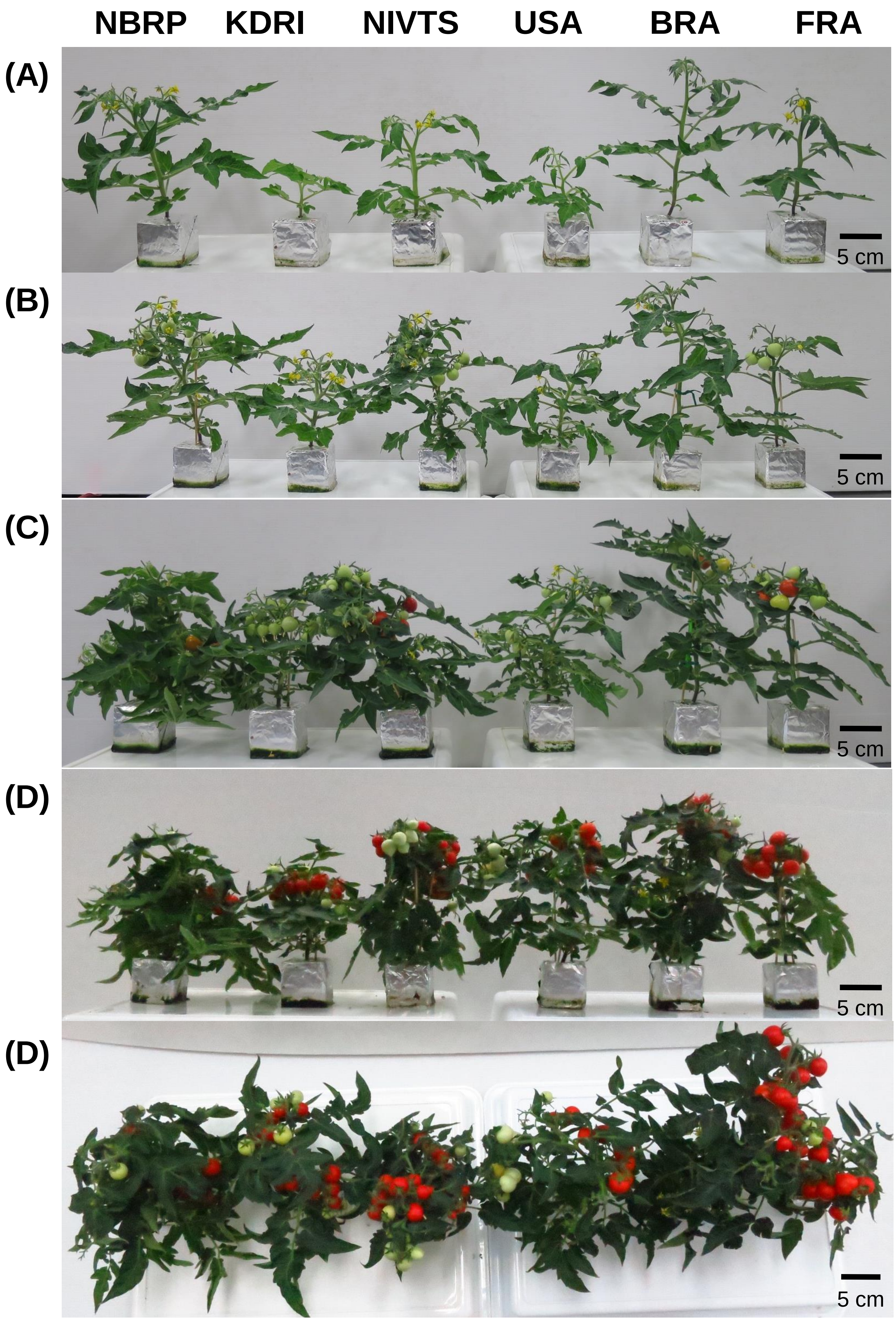

Figure SD1-2. Continuous phenotypic alteration of six Micro-Tom lines (NBRP, KDRI, NIVTS, USA, BRA, and FRA) in cultivation room.

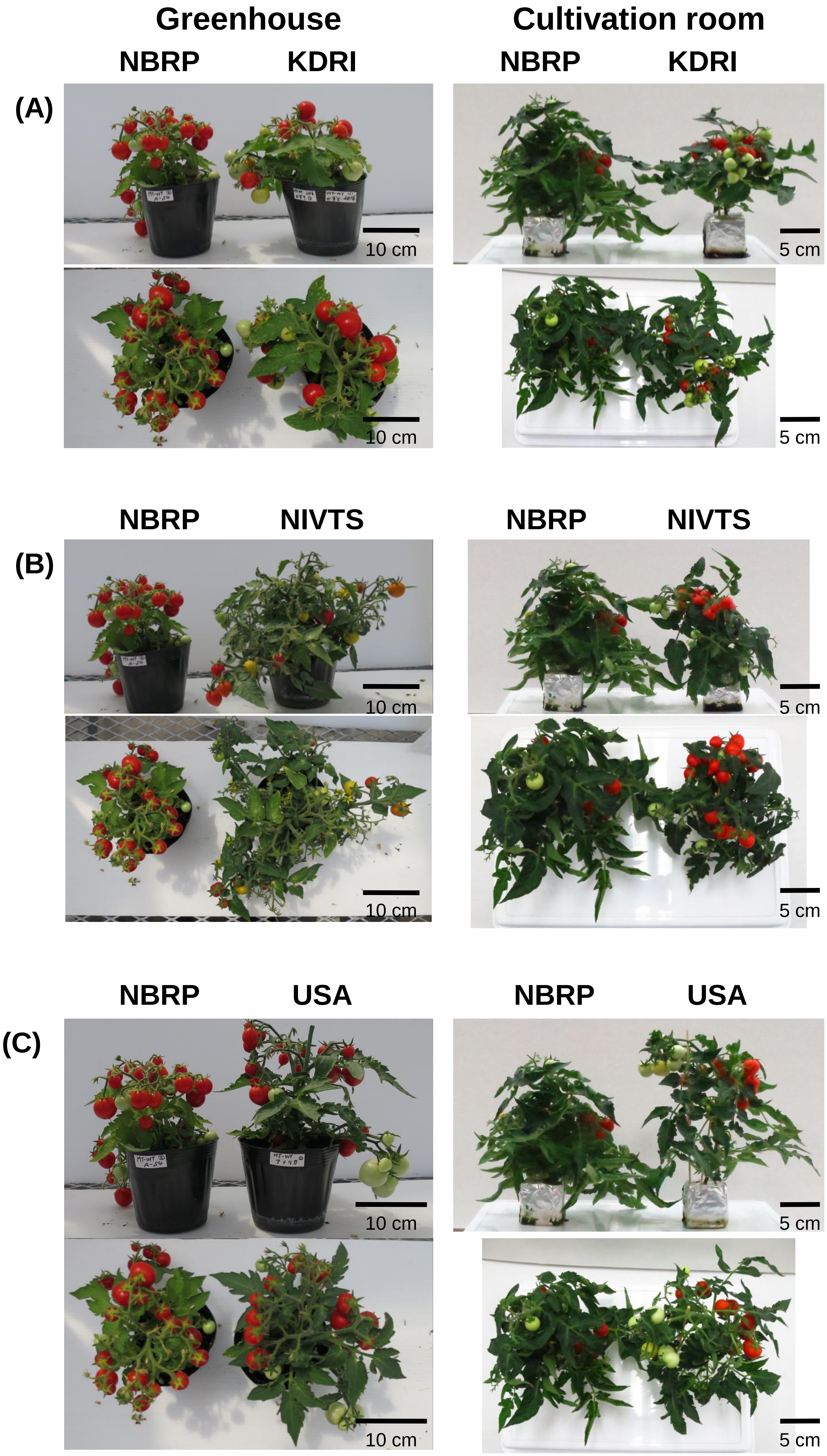

Figure SD1-3. Phenotypic difference between NBRP line and five Micro-Tom lines (KDRI, NIVTS, USA, BRA, and FRA) in a greenhouse (left) and cultivation room (right).

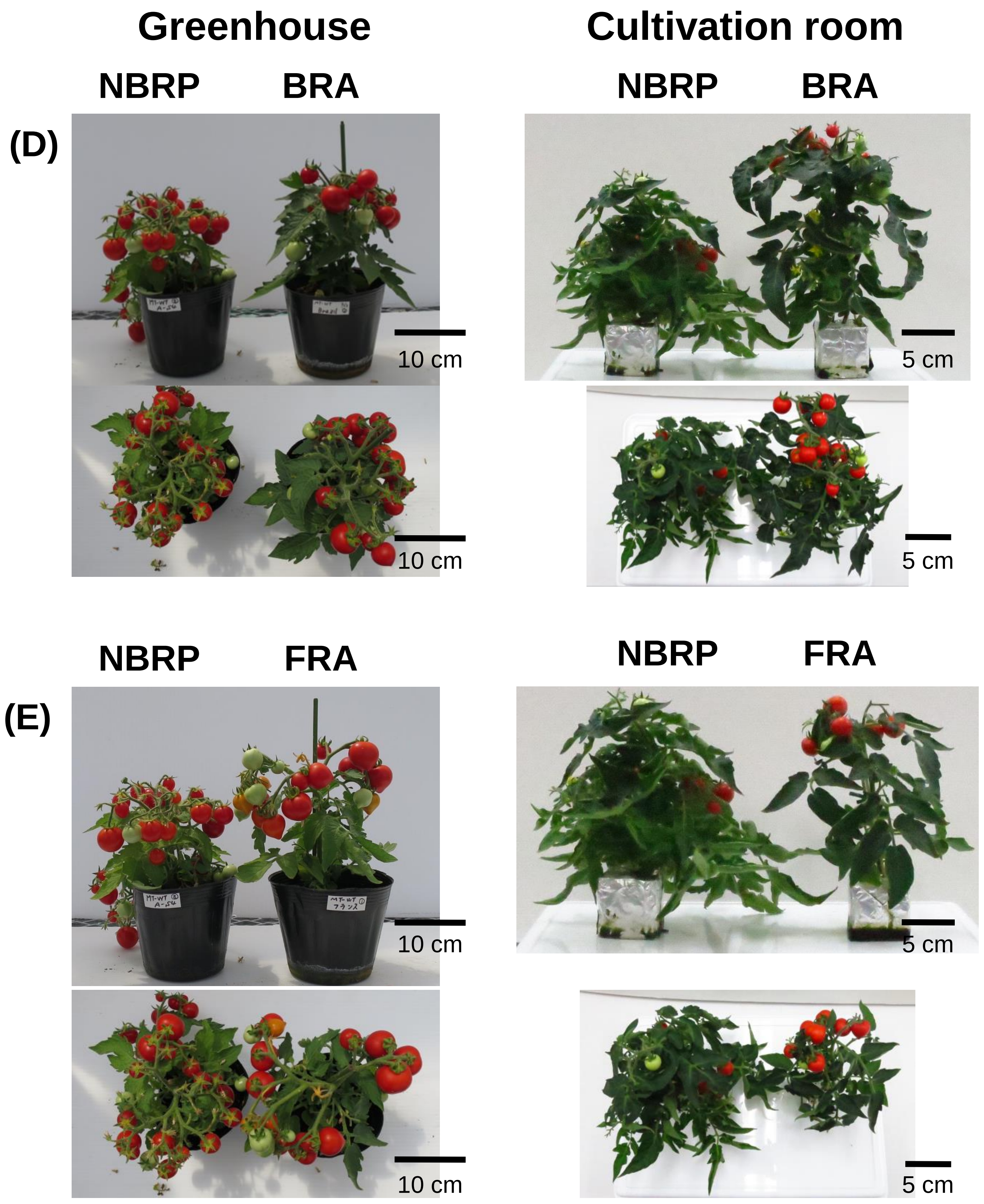
Greenhouse
Cultivation room
NBRP
BRA
NBRP
BRA
(D)
10 cm
5 cm
10 cm
5 cm
NBRP
FRA
NBRP
FRA
(E)
10 cm
5 cm
10 cm
5 cm

Figure SD1-3.

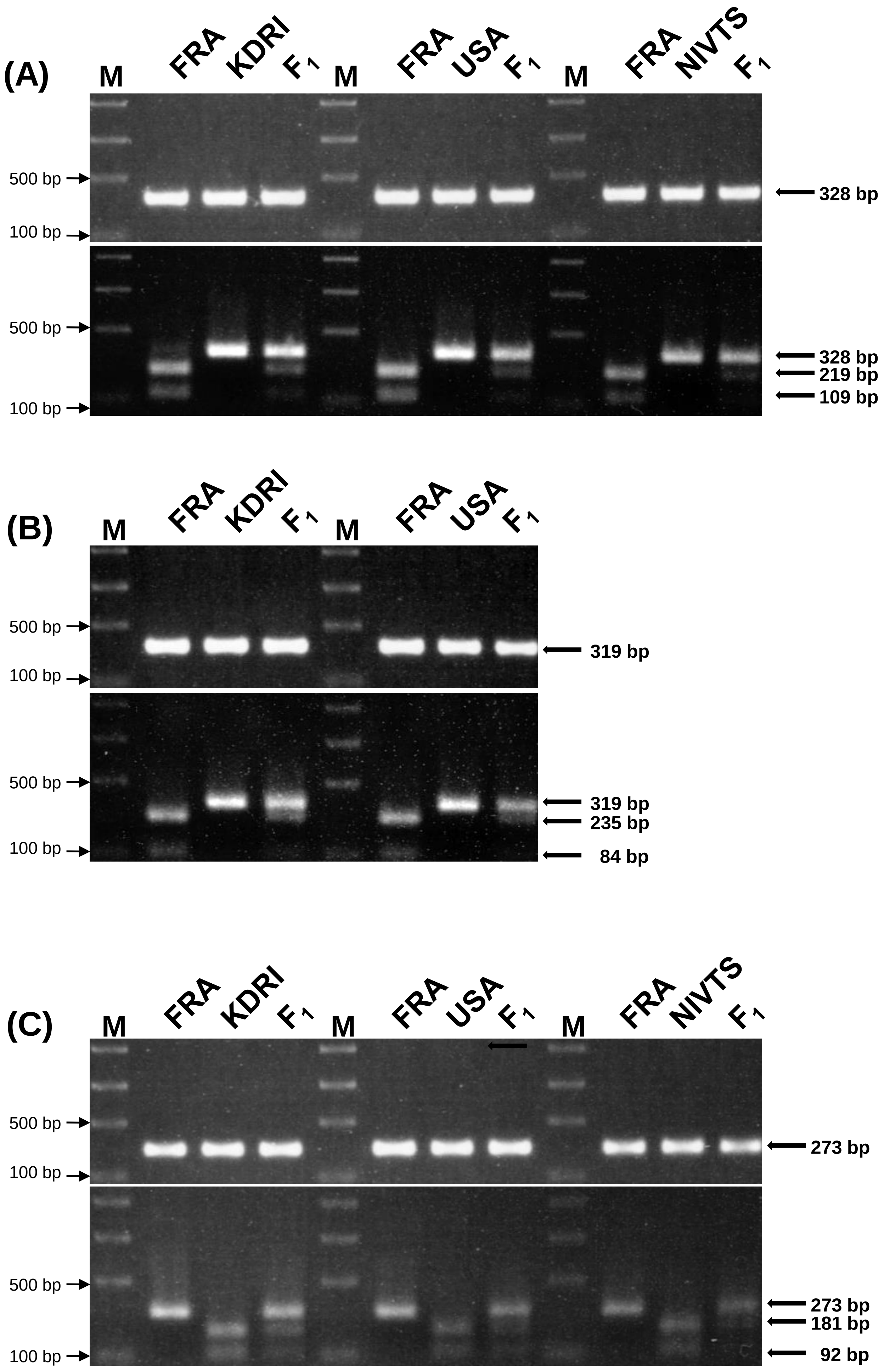

Figure SD1-4. Detection of genetic polymorphism between FRA line and four Micro-Tom lines (KDRI, NIVTS, USA, and BRA) using CAPs markers.

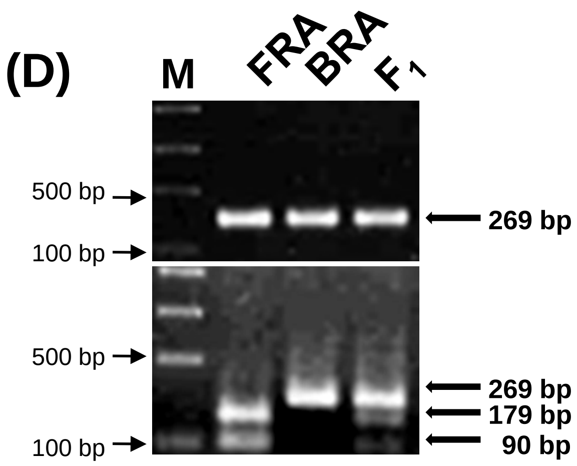
(D)
M
FRA
BRA
F 1
500 bp
100 bp
269 bp
500 bp
100 bp
269 bp
179 bp
90 bp
(E)
M
FRA
BRA
F 1
500 bp
100 bp
345 bp
500 bp
100 bp
345 bp
238 bp
107 bp

Figure SD1-4.

Figure legends

Figure SD1-1. Continuous phenotypic alteration of six Micro-Tom lines (NBRP, KDRI, NIVTS, USA, BRA, and FRA) in a greenhouse.

The photos were taken on Apr 25, 2018 (A), May 23, 2018 (B), Jun 11, 2018 (C), and Jun 25, 2018 (D). The photo (E) showed from above on Jun 25, 2018. All harvested fruits were collected from each plant on Jun 25, 2018 (F).

Figure SD1-2. Continuous phenotypic alteration of six Micro-Tom lines (NBRP, KDRI, NIVTS, USA, BRA, and FRA) in cultivation room.

The photos were taken on May 7, 2018 (A), May 25, 2018 (B), Jun 14, 2018 (C), and Jul 5, 2018 (D). The photo (E) showed from above on Jul 5, 2018.

Figure SD1-3. Phenotypic difference between NBRP line and five Micro-Tom lines (KDRI, NIVTS, USA, BRA, and FRA) in a greenhouse (left) and cultivation room (right).

The photos from the front (upper row) and above (lower row) showed phenotypic variation in a greenhouse (left) and cultivation room (right) between NBRP line and five Micro-Tom lines: KDRI (A), NIVTS (B), USA (C), BRA (D), and FRA (E).

Figure SD1-4. Detection of genetic polymorphism between FRA line and four Micro-Tom lines (KDRI, NIVTS, USA, and BRA) using CAPs markers.

To evaluate genetic polymorphism between five Micro-Tom lines (KDRI, NIVTS, USA, BRA, and FRA), CAPs makers were developed using five Micro-Tom lines and F_1 lines crossed by FRA line and four others (KDRI, NIVTS, USA, and BRA). As representative CAPs markers in this study, some electrophoresis results were shown using each primer NO. 1 (A), NO. 2 (B), NO. 12 (C), NO. 16 (D), and NO. 11 (E), following Supplementary Data1Table-1. Each DNA fragment (upper) was amplified by each CAPs maker and was digested with each restriction enzyme (lower), following Supplementary Data1Table-2. Lane M shows gene ladder fast 1 (Nippon gene, Tokyo, Japan). Lane F_1 shows each F_1 hybrid between FRA line and each Micro-Tom line.
